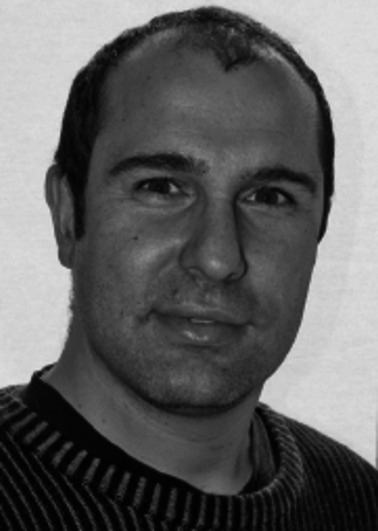# Organocatalytic Strategies for the Development of the Enantioselective Inverse‐electron‐demand Hetero‐Diels‐Alder Reaction

**DOI:** 10.1002/chem.202101696

**Published:** 2021-07-13

**Authors:** Víctor Laina‐Martín, Jose A. Fernández‐Salas, José Alemán

**Affiliations:** ^1^ Departamento de Química Orgánica (módulo 1) Facultad de Ciencias Universidad Autónoma de Madrid 28049- Madrid Spain; ^2^ Institute for Advanced Research in Chemical Sciences (IAdChem) Universidad Autónoma de Madrid 28049- Madrid Spain

**Keywords:** asymmetric synthesis, cycloadditions, Diels-Alder, heterocycles, organocatalysis

## Abstract

Cycloaddition reactions, in particular Diels‐Alder reactions, have attracted a lot of attention from organic chemists since they represent one of the most powerful methodologies for the construction of carbon‐carbon bonds. In particular, inverse‐electron‐demand hetero‐Diels‐Alder reactions have been an important breakthrough for the synthesis of heterocyclic compounds. Among all their variants, the organocatalytic enantioselective version has been widely explored since the asymmetric construction of diversely functionalized scaffolds under reaction conditions encompassed within the green chemistry field is of great interest. In this review, a profound revision on the latest advances on the organocatalytic asymmetric inverse‐electron demand hetero‐Diels‐Alder reaction is shown.

## Graphical abstract

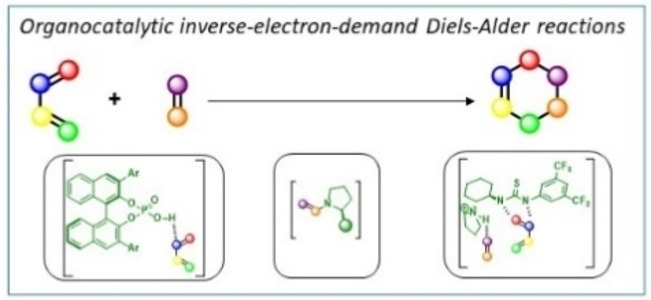

## 1 Introduction^[1]^

**Scheme 1:**
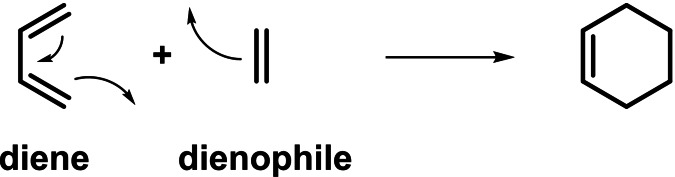
General outcome for a Diels‐Alder reaction.

A cycloaddition reaction is a reaction in which two or more unsaturated molecules (or parts of the same molecule) merge with the formation of a cyclic adduct in which there is a net reduction of the bond multiplicity, generating structural diversity in a single operation. Among all the cycloaddition reactions described during the last decades, Diels‐Alder reaction has become the most useful and exploited one in modern organic chemistry.^[2]^ It implies the reaction between a dienophile, which contributes with two electrons to the reaction, and a conjugated diene, which contributes with four electrons, leading to a six‐membered ring (Scheme 1).

**Figure 1:**
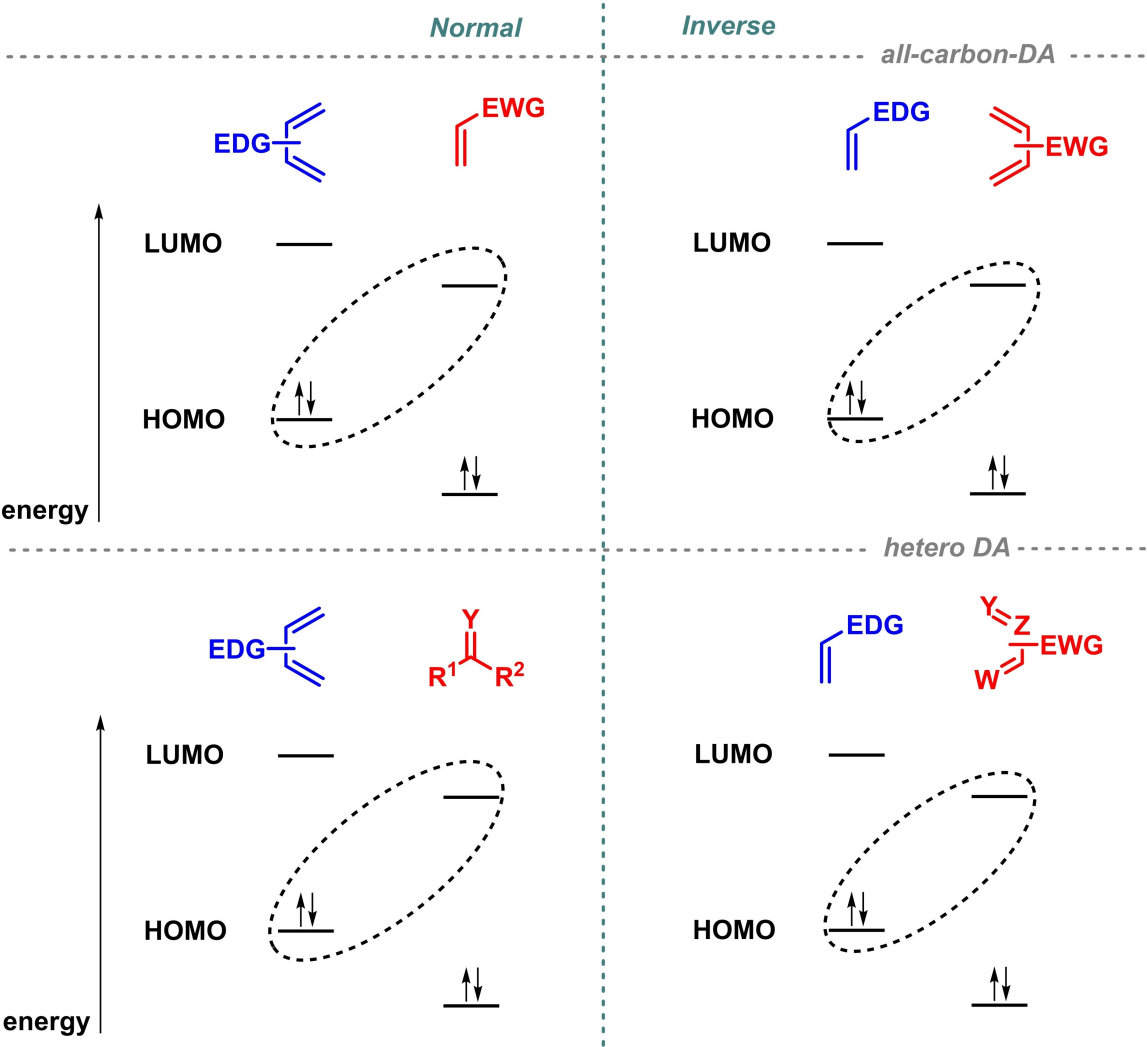
Normal‐demand and inverse‐electron‐demand all‐carbon and hetero Diels‐Alder reactions.

In general, Diels‐Alder reactions can be classified in two different types according to the relative energies of the highest occupied molecular orbital (HOMO) and the lowest unoccupied molecular orbital (LUMO) of both the diene and the dienophile based on the Hückel molecular orbital model. In this sense, normal‐demand Diels‐Alder reaction implies the interaction between the HOMO of the diene and the LUMO of the dienophile. Thus, it involves the reaction between electron‐rich dienes and electron‐poor dienophiles (Figure 1, top‐left). However, inverse‐electron‐demand Diels‐Alder reaction (IEDDAR) implies the interaction between the LUMO of the diene and the HOMO of the dienophile. As a result, it requires the reaction between electron‐poor dienes and electron‐rich dienophiles (Figure 1, top‐right).[^2^, ^3^]

**Figure 2:**
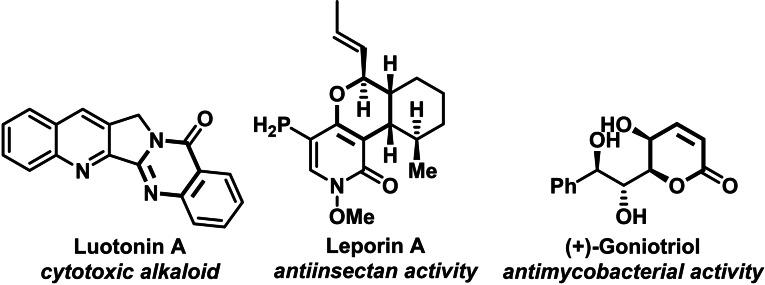
Selected examples of biologically active compounds in whose syntheses an IEDHDAR represents a key step.

Of special interest are inverse‐electron‐demand hetero‐Diels‐Alder reaction (IEDHDAR) variants, as they allow the incorporation of the functional group into the ring.^[3]^ In this case, the reaction is governed by the interaction between the LUMO of an electron‐poor hetero diene and the HOMO of the dienophile (Figure 1, bottom‐right). In this sense, IEDHDAR has opened the possibility to synthesize in a single operation a wide variety of heterocycles, which are recognizable and prevalent cores in biologically active products and pharmaceuticals.^[4]^ Furthermore, IEDHDAR has also been employed as a key step for the synthesis of different natural products and biologically active compounds such as Luotonin A, Leporin A and Goniotriol, among others (Figure 2).^[4]^ In addition, the IEDHDA approach has been extensively used for biolabeling and the development of biorthogonal chemistry, which involves reactions that happen inside of living systems and do not interfere with biological processes.^[3]^

Additionally, the stereoselective construction of carbon‐carbon bonds still stands as a crucial objective and preserves a preferred position in the organic chemistry research.^[5]^ In this context, the development of new asymmetric IEDHDA systems has been an ongoing target within the organic chemistry field.^[6]^ Numerous studies based on different catalytic systems have been reported over the past decades pursuing this challenging objective. Among them, organocatalysis has continuously evolved as an alternative to develop asymmetric IEDHDA reactions, becoming a powerful tool for the asymmetric synthesis of a wide range of compounds during the last decades.^[6]^

**Figure 3:**
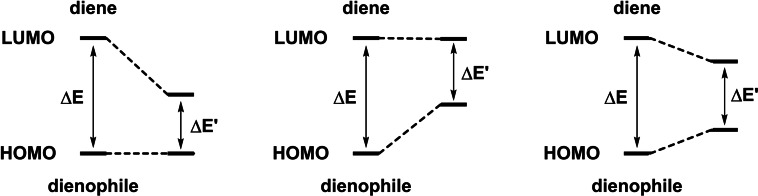
Different strategies for the catalytic activation of both reaction partners in an IEDHDAR.

Since the appearance of organocatalysis, several research groups have targeted this exciting objective for the development of different activation modes in order to embrace a wide number of asymmetric IEDHDA systems, synthesizing a plethora of heterocyclic compounds in an enantioselective fashion.^[6]^ In this regard, three main strategies have been followed: i) LUMO‐lowering strategies, ii) HOMO‐raising approaches, and iii) strategies based on both a LUMO‐lowering and a HOMO‐raising activation (Figure 3).

In this regard, a revision on the latest advances made on organocatalytic asymmetric inverse‐electron demand hetero‐Diels‐Alder methods, considering the main strategies employed for that purpose, has been addressed in the present review.

## 2 LUMO‐Lowering Strategies

**Scheme 2:**
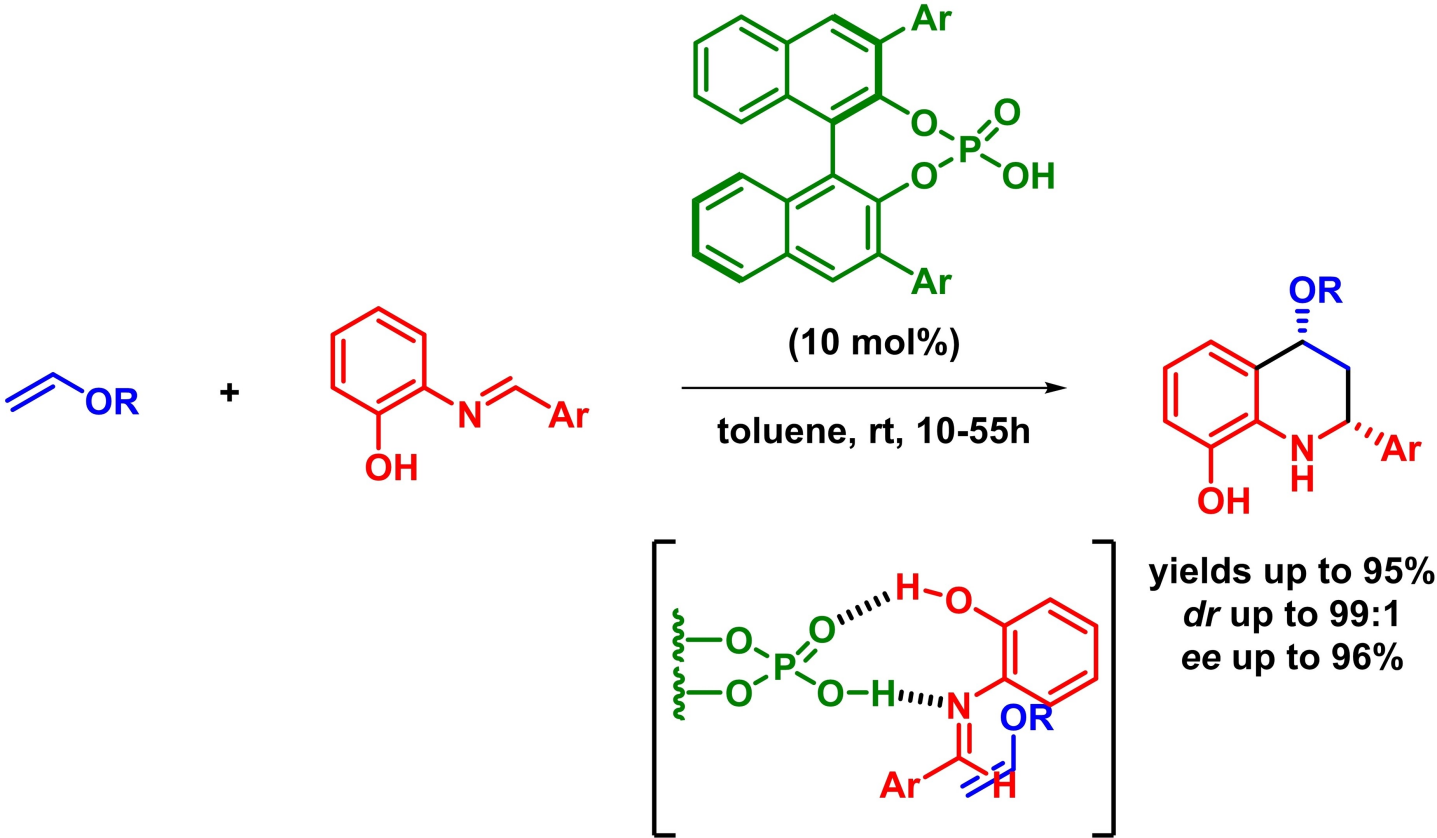
Asymmetric synthesis of tetrahydroquinoline derivatives via IEDHDAR.

The use of Brønsted acid catalysts has been widely explored in asymmetric synthesis.^[7]^ Among all the interesting systems based on organocatalysis, phosphoric acids have demonstrated their capability to activate different dienes, giving rise to an interesting LUMO‐lowering strategy for the construction of enantiopure heterocycles through IEDHDA reactions. In this context, Akiyama reported the enantioselective synthesis of tetrahydroquinoline derivatives via an asymmetric inverse‐electron‐demand *aza*‐Diels‐Alder reaction. The reaction takes place in high yields, excellent diastereoselectivities and high enantioselectivities in the presence of different aldimines and electron‐rich alkenes (Scheme 2). Moreover, the authors claim that the reaction proceeds through a nine‐membered cyclic transition state, where the oxygen of the phosphoric acid forms a hydrogen bond with the hydrogen of the hydroxyl group (LUMO_diene_‐lowering), permitting the nucleophilic attacking from the *Re* face of the imine.^[8]^

**Scheme 3:**
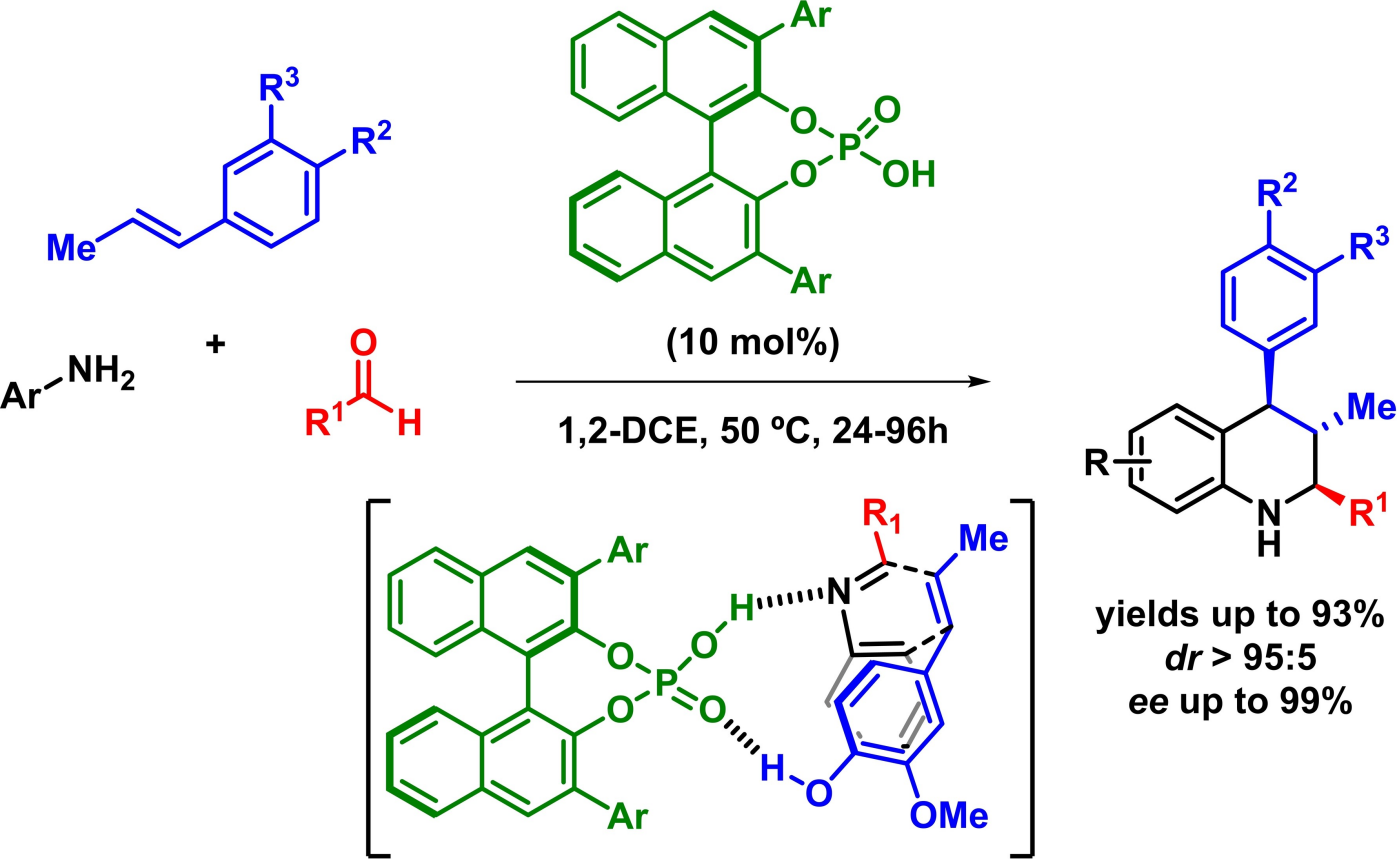
Asymmetric synthesis of 2,3,4‐trisubstituted tetrahydroquinolines via phosphoric acid‐catalyzed Povarov reaction.

Taking advantage of previous reports,[^8^, ^9^] Masson and coworkers reported the enantioselective synthesis of 2,3,4‐trisubstituted tetrahydroquinolines through an asymmetric inverse‐electron‐demand *aza*‐Diels‐Alder reaction between isoeugenol derivatives and in situ formed imines. The reaction proceeds with excellent yields, diastereoselectivities and enantioselectivities (Scheme 3).^[10]^ The authors propose that the ability of the phosphoric acid to form H‐bonds with the phenol (demonstrated to be crucial for the reaction) and the imine would explain the stereochemistry of the reaction.

**Scheme 4:**
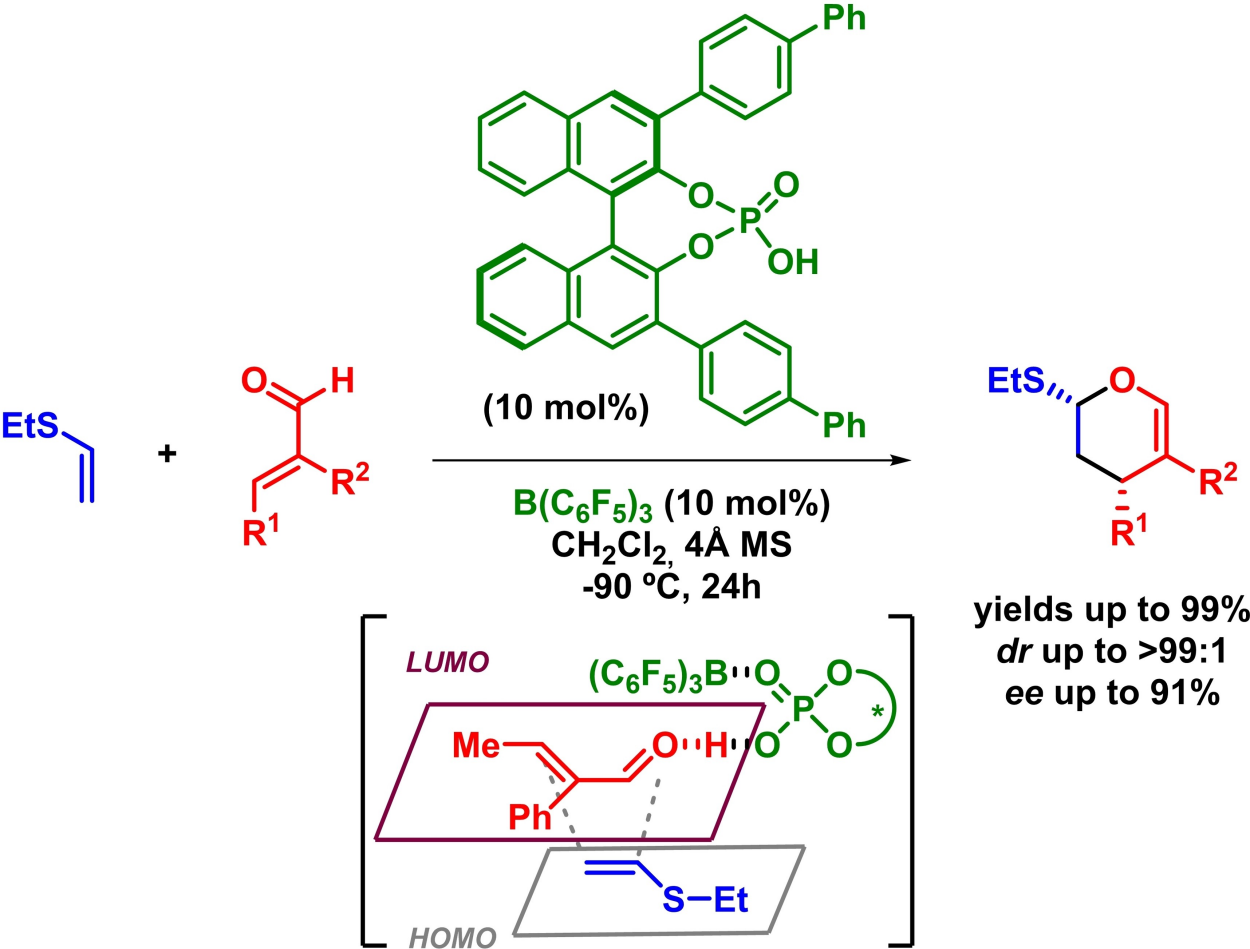
Enantioselective IEDHDAR between acroleins and ethyl vinyl sulfide.

Very recently, Ishihara and coworkers have described a LUMO‐lowering enantioselective inverse‐electron‐demand *oxa*‐Diels‐Alder reaction by activation of acroleins. The reaction takes place with good yields, diastereoselectivities and enantioselectivities in the presence of differently substituted acroleins and ethyl vinyl sulfide (Scheme 4). Furthermore, the authors propose that the reaction undergoes through an *endo* orbital reaction pathway via the in situ formation of a chiral supramolecular Brønsted acid catalyst activated by boron‐based Lewis acid.^[11a]^

## 3 HOMO‐Raising Strategies

The activation of the HOMO of different dienophiles has been the alternative organocatalytic strategy that has allowed the development of new IEDHDA systems. Amine‐based (enamine and dienamine) catalysis and N‐heterocyclic carbenes have been the systems of choice as they can covalently generate the dienophile in situ, enhancing the energy of the HOMO of different aldehydes through the formation of the transient chiral (di)enamines or enolates, respectively, which permit the corresponding cycloaddition with an excellent enantiocontrol.[^6a^, ^6d^]

**Scheme 5:**
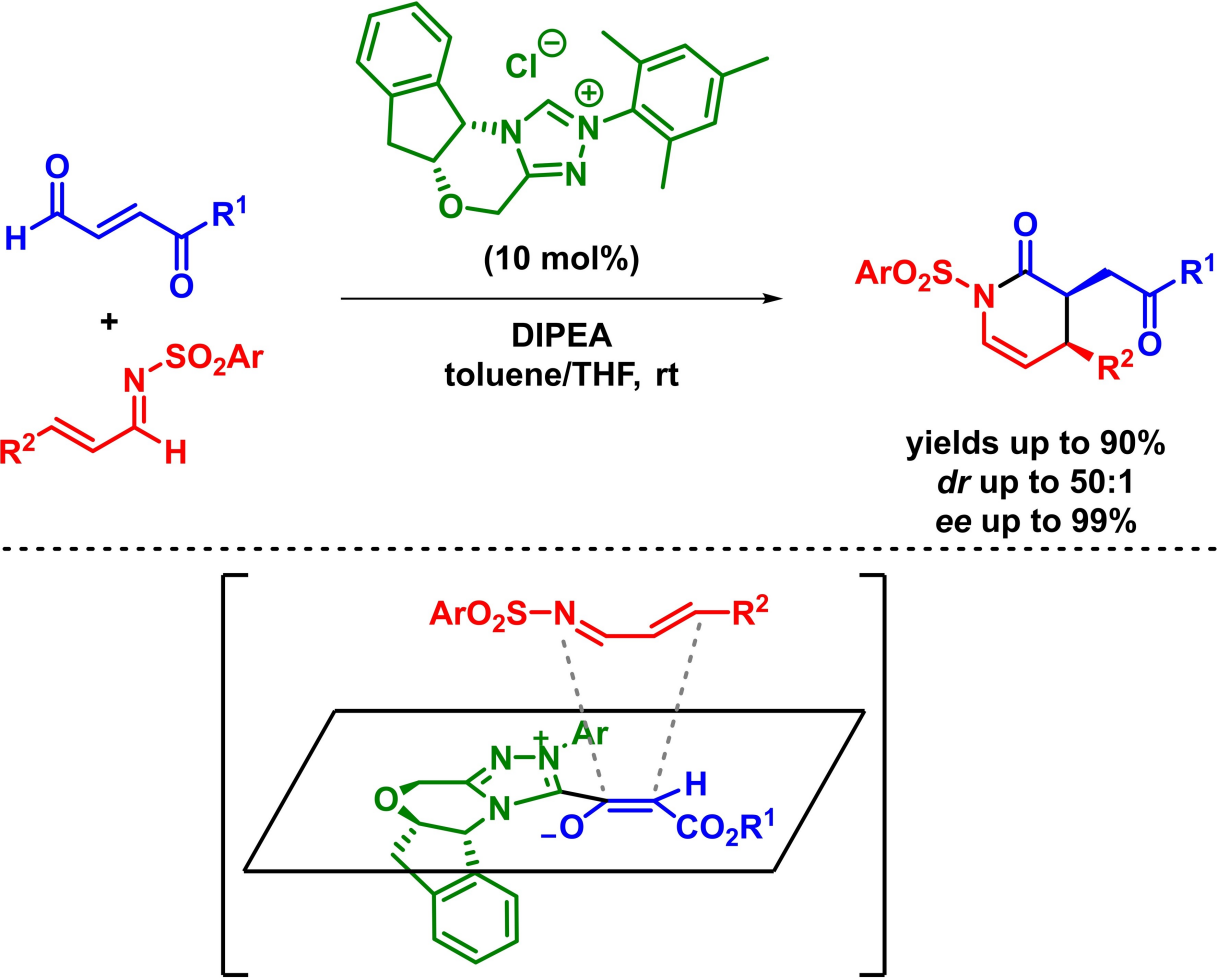
NHC‐catalyzed asymmetric synthesis of dihydropyridinone derivatives via IEDHDAR.

In this context, Bode and coworkers reported the NHC‐catalyzed^[12]^ asymmetric synthesis of dihydropyridinone derivatives via an enantioselective inverse‐electron‐demand *aza*‐Diels‐Alder reaction. This represented the first example of an NHC‐catalyzed generation of highly reactive dienophiles that participates in an IEDHDAR with highly reactive *α*,*β*‐unsaturated imines. The reaction takes place in high yields and excellent diastereoselectivities and enantioselectivities for a wide range of starting materials (Scheme 5). Additionally, the authors argue that the reaction mechanism proceeds through an *endo*‐selective concerted mechanism in which only the resulting (*Z*)‐enolate reacts, leading to the observed absolute configuration of the final products.^[13]^

**Scheme 6:**
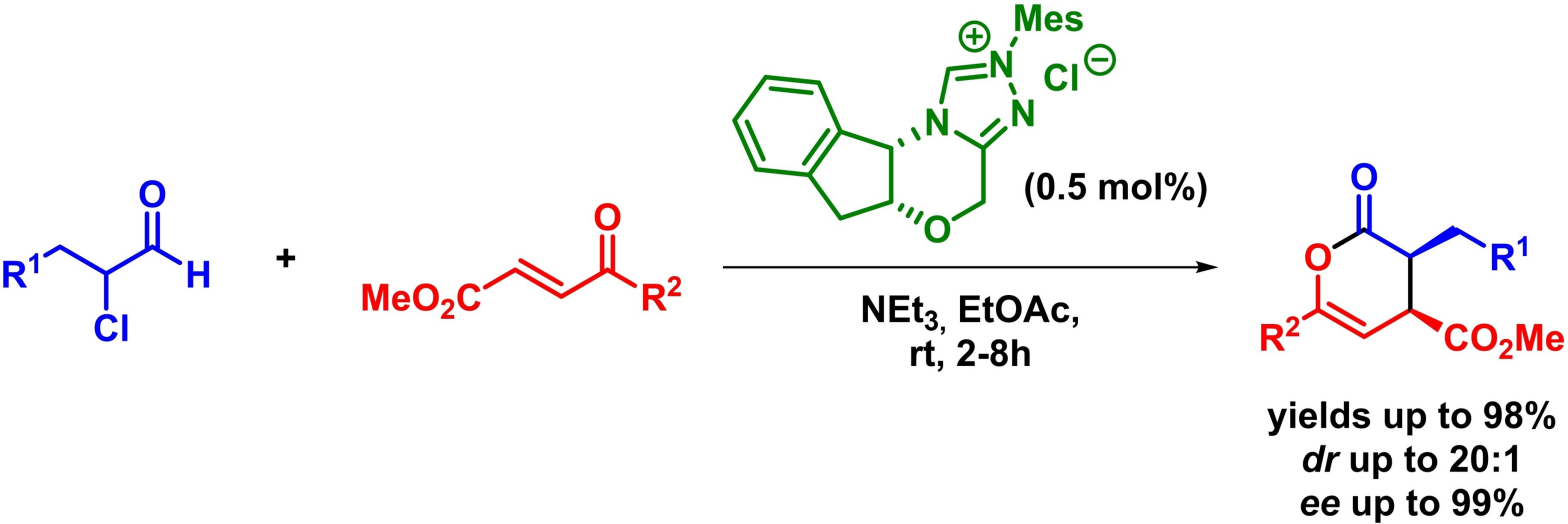
NHC‐catalyzed enantioselective synthesis of dihydropyranones via IEDHDAR.

In a similar way, Bode extended this strategy to the asymmetric synthesis of highly substituted dihydropyran‐2‐ones. The reaction takes place in the presence of very low amounts of an NHC organocatalyst and with excellent yields, diastereoselectivities and enantioselectivities (Scheme 6).^[14]^

**Scheme 7:**
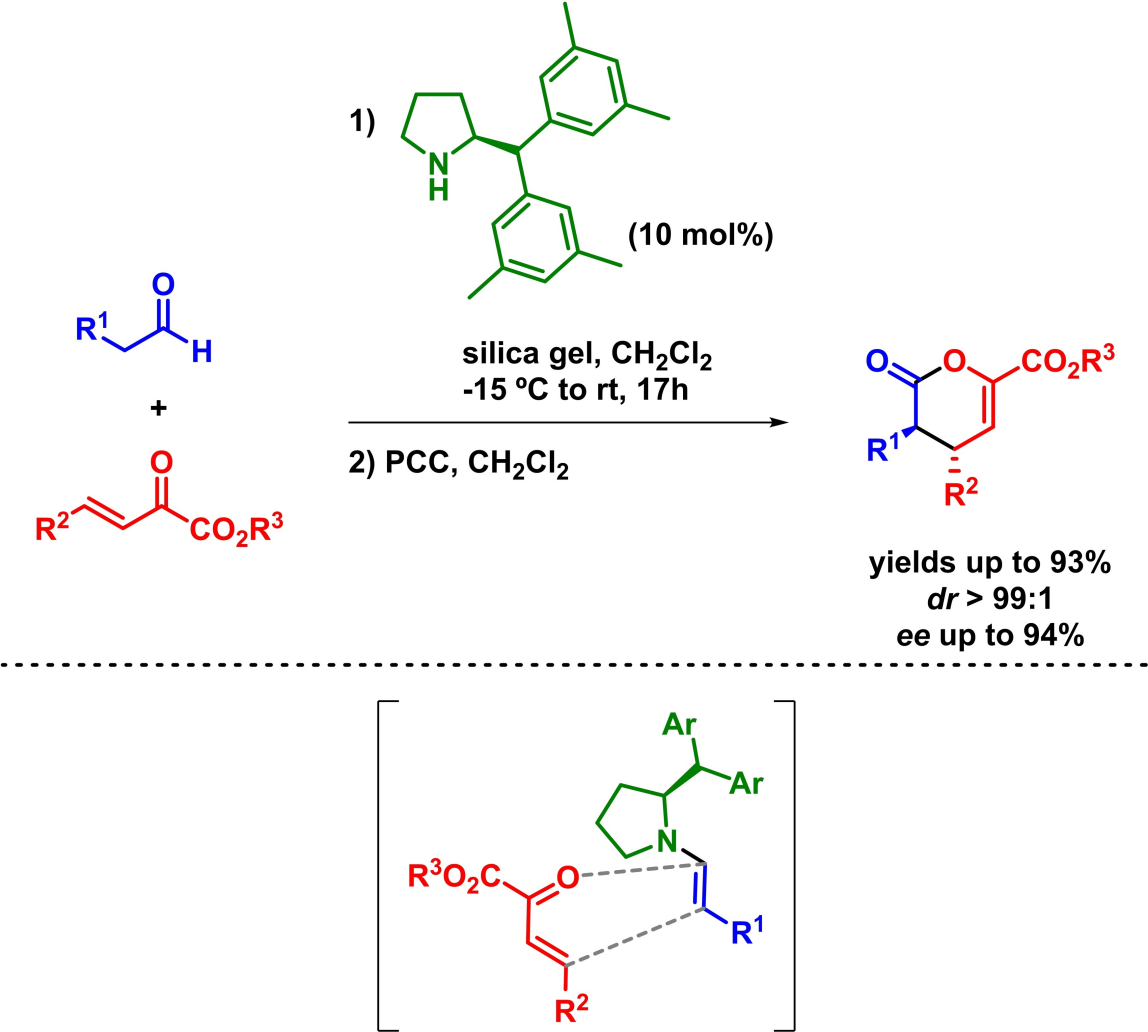
First example of an organocatalytic asymmetric IEDHDAR.

On the other hand, the formation of enamine or dienamine intermediates (chiral dienophiles) through amine catalysis has been the most explored alternative for the HOMO‐raising approach in the enantioselective organocatalytic inverse‐electron‐demand hetero‐Diels‐Alder reaction methodology. In relation to the enamine strategy, Jørgensen and coworkers reported the first organocatalytic asymmetric IEDHDAR. The reaction takes place in excellent yields and enantioselectivities (Scheme 7). In addition, it is worth to mention that a PCC‐mediated oxidation of the hemiacetal is required to obtain good levels of diastereoselectivity in the reaction.^[15]^

**Scheme 8:**
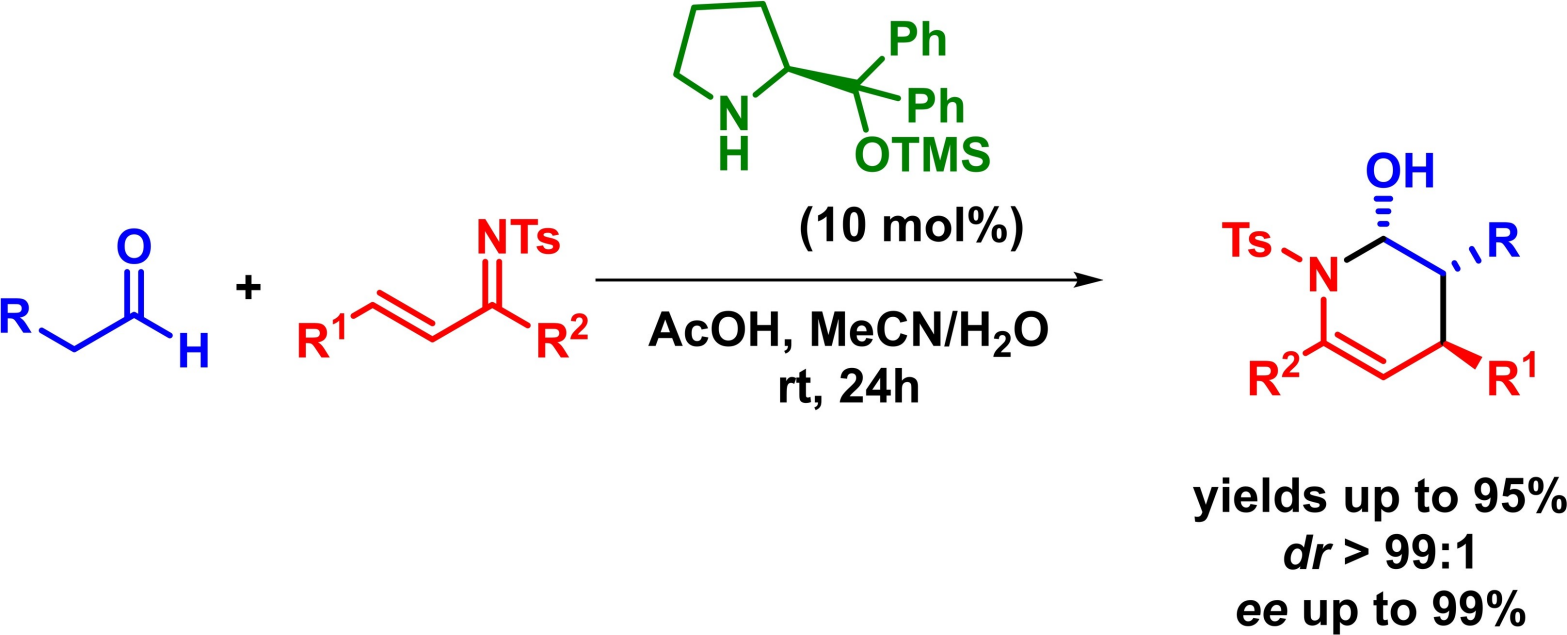
Amine‐catalyzed enantioselective IEDHDAR between *N*‐sulfonyl‐1‐aza‐1,3‐butadienes and aliphatic aldehydes.

Based on a comparable strategy, Chen reported the asymmetric inverse‐electron‐demand *aza*‐Diels‐Alder reaction between *N*‐sulfonyl‐1‐aza‐1,3‐butadienes and differently substituted linear aliphatic aldehydes. The reaction proceeds with excellent yields, diastereoselectivities and enantioselectivities in the presence of low catalyst loadings (Scheme 8). Moreover, hemiaminals are obtained with high diastereoselectivities without the need of an additional oxidation step.^[16]^

**Scheme 9:**
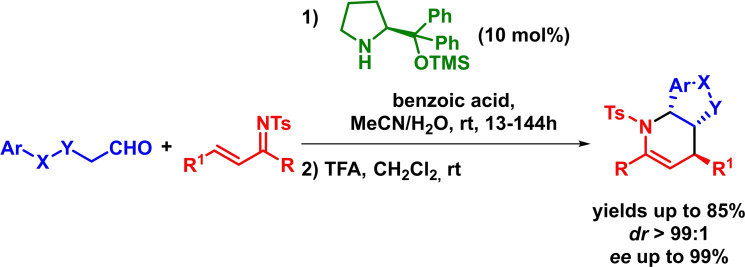
Asymmetric synthesis of fused piperidine derivatives via IEDHDA and Friedel Crafts reaction sequence.

In 2011, and taking advantage of their preceding report,^[16]^ Chen and coworkers described the enantioselective synthesis of fused piperidine frameworks through an asymmetric inverse‐electron‐demand *aza*‐Diels‐Alder and Friedel–Crafts reaction sequence. The reaction takes place in excellent yields, diastereoselectivities and enantioselectivities in the presence of a wide range of different starting materials (Scheme 9).^[17]^

**Scheme 10:**
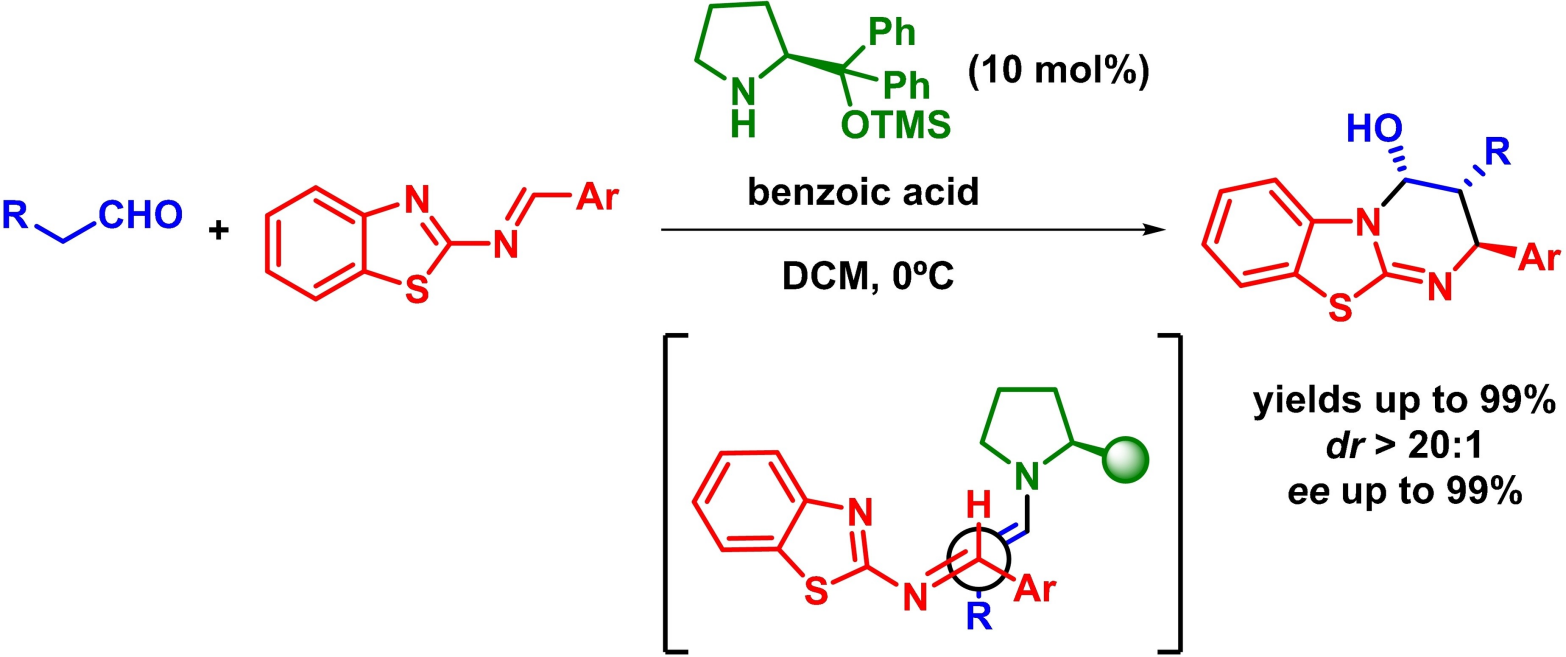
Organocatalytic asymmetric synthesis of benzothiazolopyrimidines via [4+2] cyclization of 2‐benzothiazolimines and aldehydes.

Very recently, Xiong and coworkers have reported an enantioselective inverse‐electron‐demand *aza*‐Diels‐Alder of 2‐benzothiazolimines as dienophiles for the asymmetric synthesis of benzothiazolopyrimidines, which are recognizable building blocks. The reaction proceeds with excellent yields, diastereoselectivities and enantioselectivities (Scheme 10).^[18]^ The nucleophilic attack of the in situ formed enamine intermediate reacts with 2‐benzothiazolimine through its *Re*‐face.

**Scheme 11:**
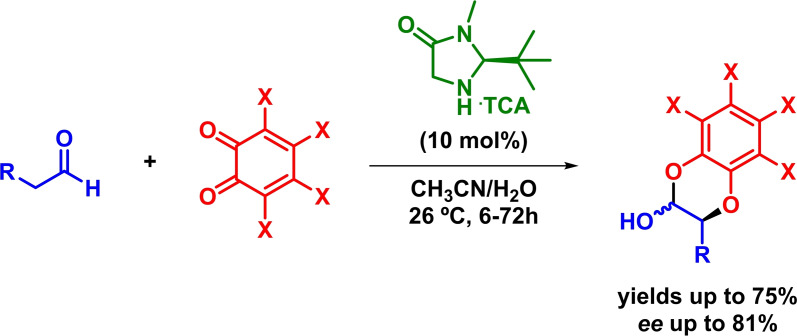
Asymmetric IEDHDAR between aliphatic aldehydes and *o*‐quinones.

Additionally, Dixon also reported an organocatalytic asymmetric inverse‐electron‐demand hetero‐Diels‐Alder reaction by facing in situ generated enamine intermediates and *o*‐quinones. The reaction takes place in the presence of an imidazolidinone‐based catalyst and with moderate to good yields and enantioselectivities (Scheme 11).^[19]^ In a similar way, Chen and coworkers described the enantioselective synthesis of hydroquinoxaline derivatives by means of an organocatalytic IEDHDAR.^[20]^

**Scheme 12:**
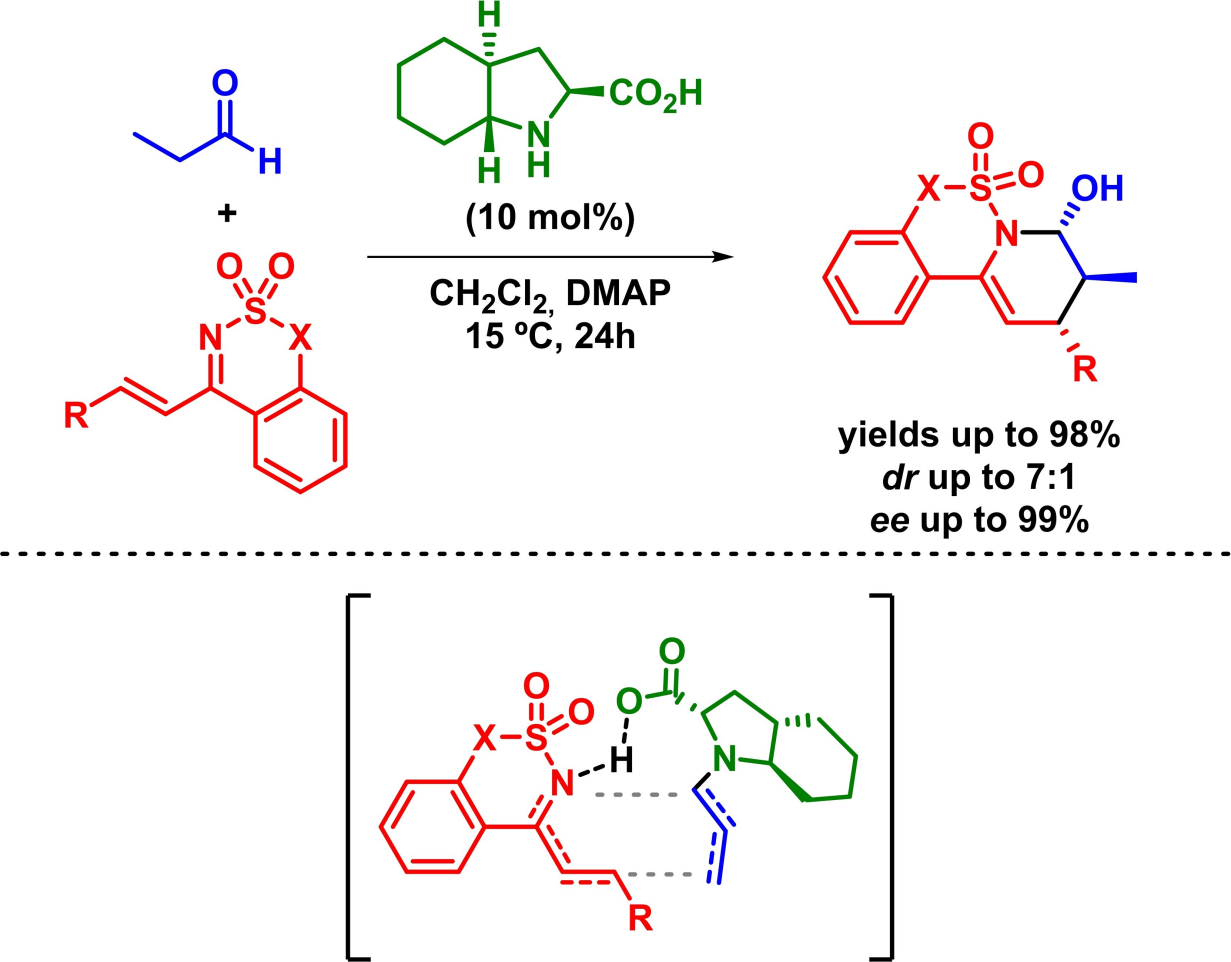
Enantioselective synthesis of dehydropiperidine derivatives through IEDHDAR.

Some years later, Liu and Zhang developed the enantioselective synthesis of dehydropiperidine derivatives via an IEDHDAR. The reaction proceeds with high yields, moderate to good diastereoselectivities and excellent enantioselectivities in the presence of different five‐ and six‐membered cyclic 1‐azadienes^[21]^ and propanal (Scheme 12).^[22]^ Furthermore, the authors suggest a concerted mechanism where the in situ formed enamine reacts with the diene. The piperidine product resulted from the top surface attack of the sulfimide to the aldehyde group, which was formed from the hydrolysis of the aminal after the cyclization.

**Scheme 13:**
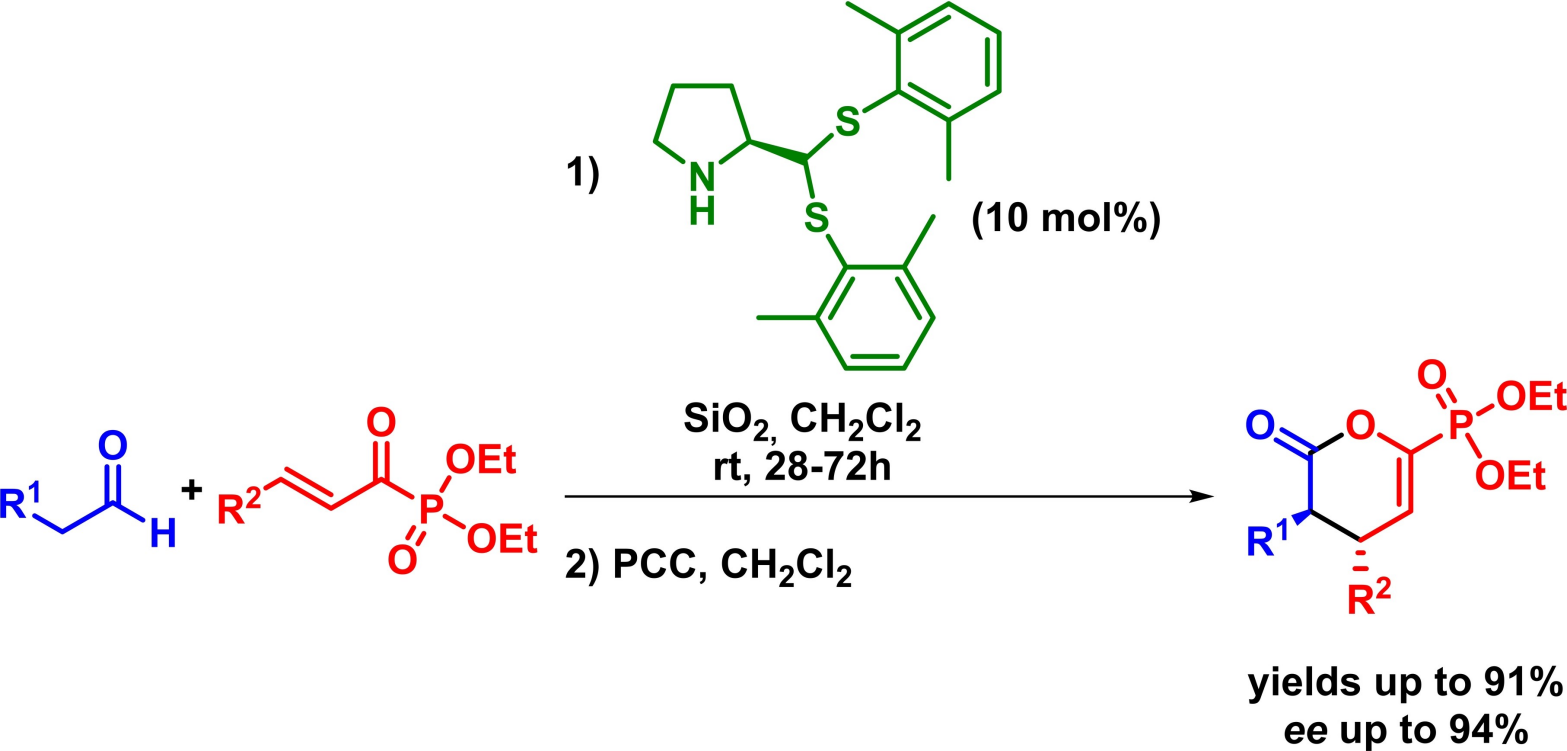
Enantioselective synthesis of 5,6‐dihydro‐*4H*‐pyran‐2‐ylphosphonates via IEDHDAR.

In addition, Zhao made also use of the same activation mode for the asymmetric synthesis of 5,6‐dihydro‐*4H*‐pyran‐2‐ylphosphonates. The reaction proceeds with high yields, moderate diastereoselectivities and good enantioselectivities in the presence of a proline‐type catalyst (Scheme 13). However, the need for an additional oxidation step is mandatory.^[23]^

**Scheme 14:**
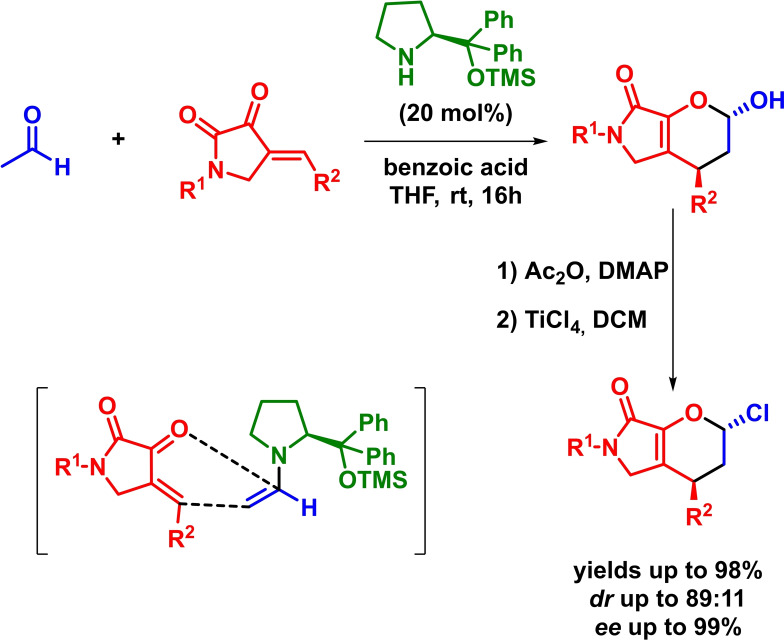
Asymmetric organocatalytic synthesis of dihydropyrans derivatives via IEDHDAR.

Contemporaneously to the previous work, Liu and co‐workers reported the asymmetric inverse‐electron‐demand hetero‐Diels‐Alder reaction between enolizable aldehydes and *α*,*β*‐unsaturated trifluoromethyl ketones in the presence of a proline and *p*‐fluorophenol as catalysts.^[24]^ Some years later, Yang, Peng and Ghou described the enantioselective synthesis of bicyclic dihydropyrans by means of an organocatalytic inverse‐electron‐demand *oxa*‐Diels‐Alder reaction in the presence of aqueous acetaldehyde (Scheme 14). The authors propose a mechanistic pathway involving an *endo*‐selective concerted mechanism in which the enamine intermediate reacts with the diene through the less sterically hindered *Si* face.^[25]^

**Scheme 15:**
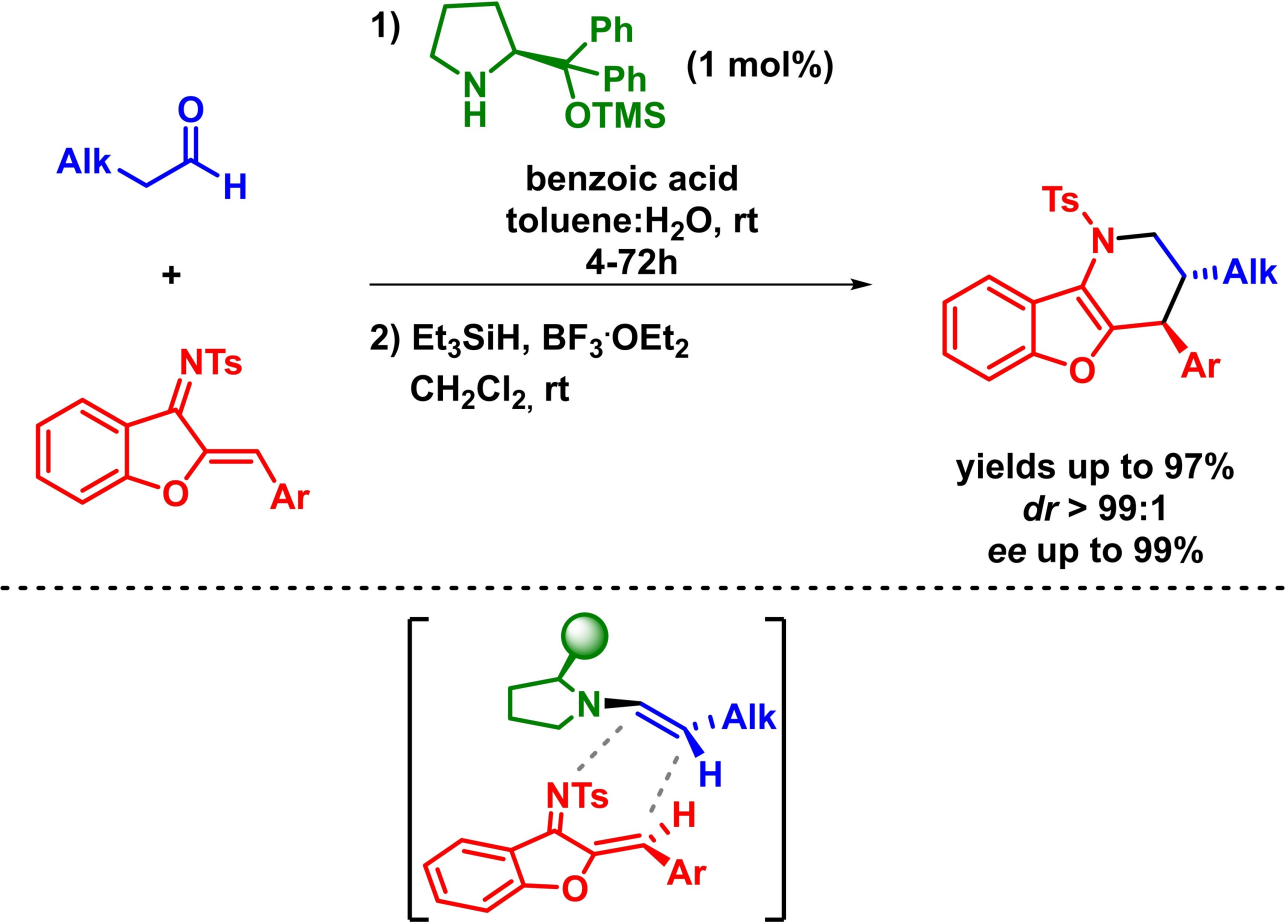
Amine‐catalyzed enantioselective synthesis of dihydropyridines via IEDHDAR.

Additionally, Zhao reported the asymmetric synthesis of dihydropyridines via IEDHDAR. The reaction takes place with excellent yields, enantioselectivities and diastereoselectivities in the presence of very low catalyst loadings. However, a reduction step is mandatory in order to obtain the dihydropyridine derivatives with excellent results since the intermediate hemiaminal species cannot be obtained in a diastereoselective manner (Scheme 15).^[26]^ Furthermore, the authors suggest a concerted mechanism of the enamine and the diene.

**Scheme 16:**
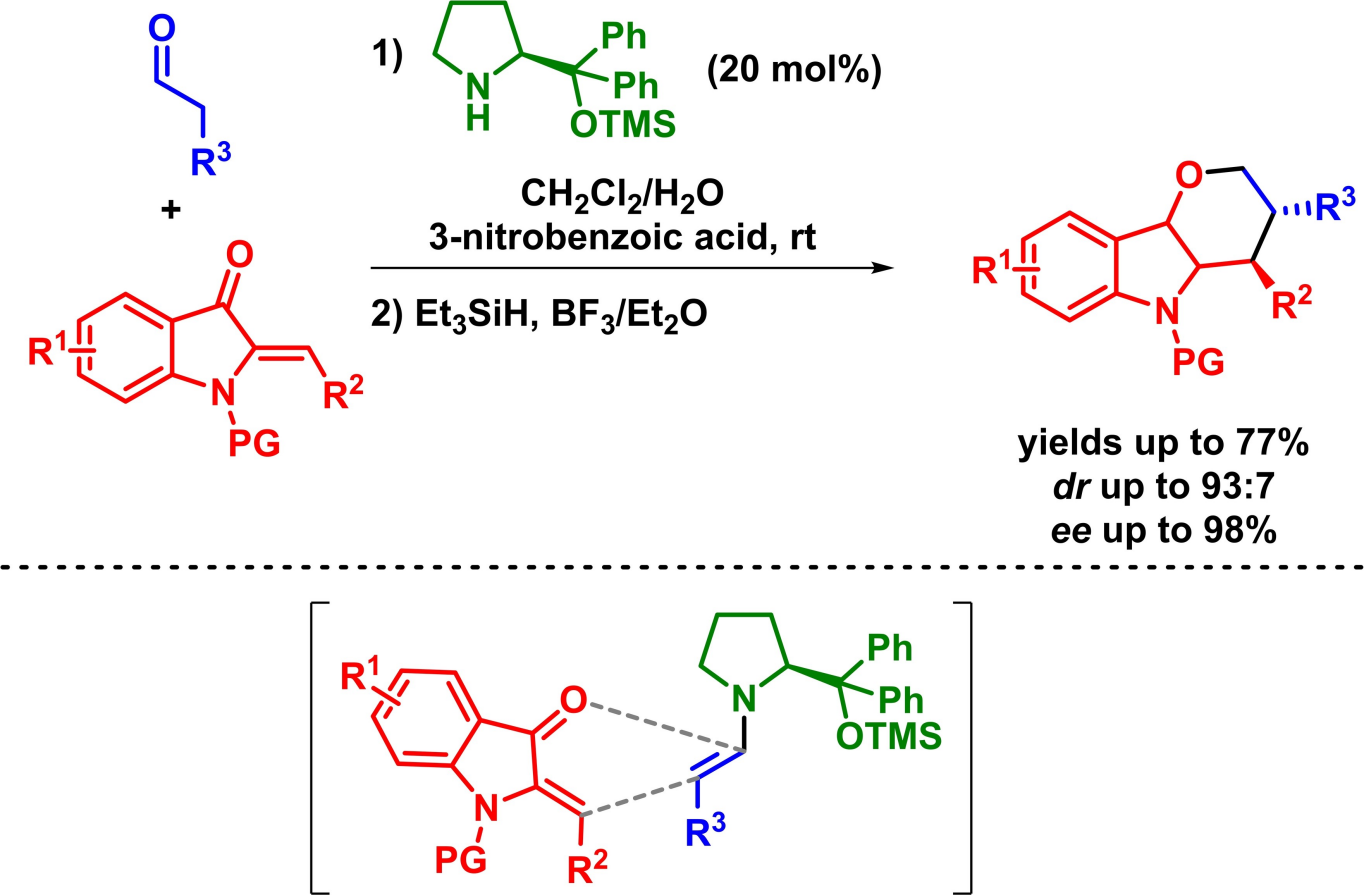
Enantioselective IEDHDAR between aldehydes and (*Z*)‐2‐ylideneoxindoles.

In a similar way, Peng and Han reported the enantioselective synthesis of hydropyrano[3,2‐*b*]indoles via an organocatalytic asymmetric inverse‐electron‐demand *oxa*‐Diels‐Alder reaction. The reaction takes place in the presence of a wide range of (*Z*)‐2‐ylideneoxindoles and aldehydes with excellent yields and enantioselectivities (Scheme 16). Nevertheless, a reduction step is again required in order to obtain the final compounds with high diastereoselectivities as the intermediate hemiacetal species cannot be obtained in a diastereoselective manner.^[27]^

**Scheme 17:**
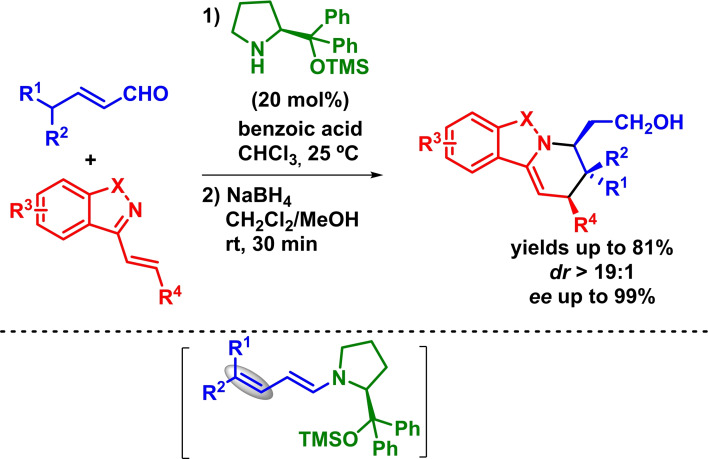
Enantioselective IEDHDAR between cyclic 1‐aza‐1,3‐butadienes and *α*,*β*‐unsaturated aldehydes.

On another hand, the use of *α*,*β*‐unsaturated aliphatic aldehydes has permitted the development of the vinylogous version of the amine‐catalyzed HOMO‐raising controlled reaction, allowing the synthesis of differently substituted heterocyclic compounds via IEDHDAR through dienamine intermediates. Thus, Chen described the asymmetric inverse‐electron‐demand *aza*‐Diels‐Alder reaction between cyclic 1‐aza‐1,3‐butadienes and *α*,*β*‐unsaturated aldehydes in the presence of a secondary amine catalyst. In this example, the regioselectivity revealed by the in situ formed dienamine shows the expected *β*,*γ*‐regioselectivity. The reaction proceeds with excellent yields, diastereoselectivities and enantioselectivities (Scheme 17).^[28]^

**Scheme 18:**
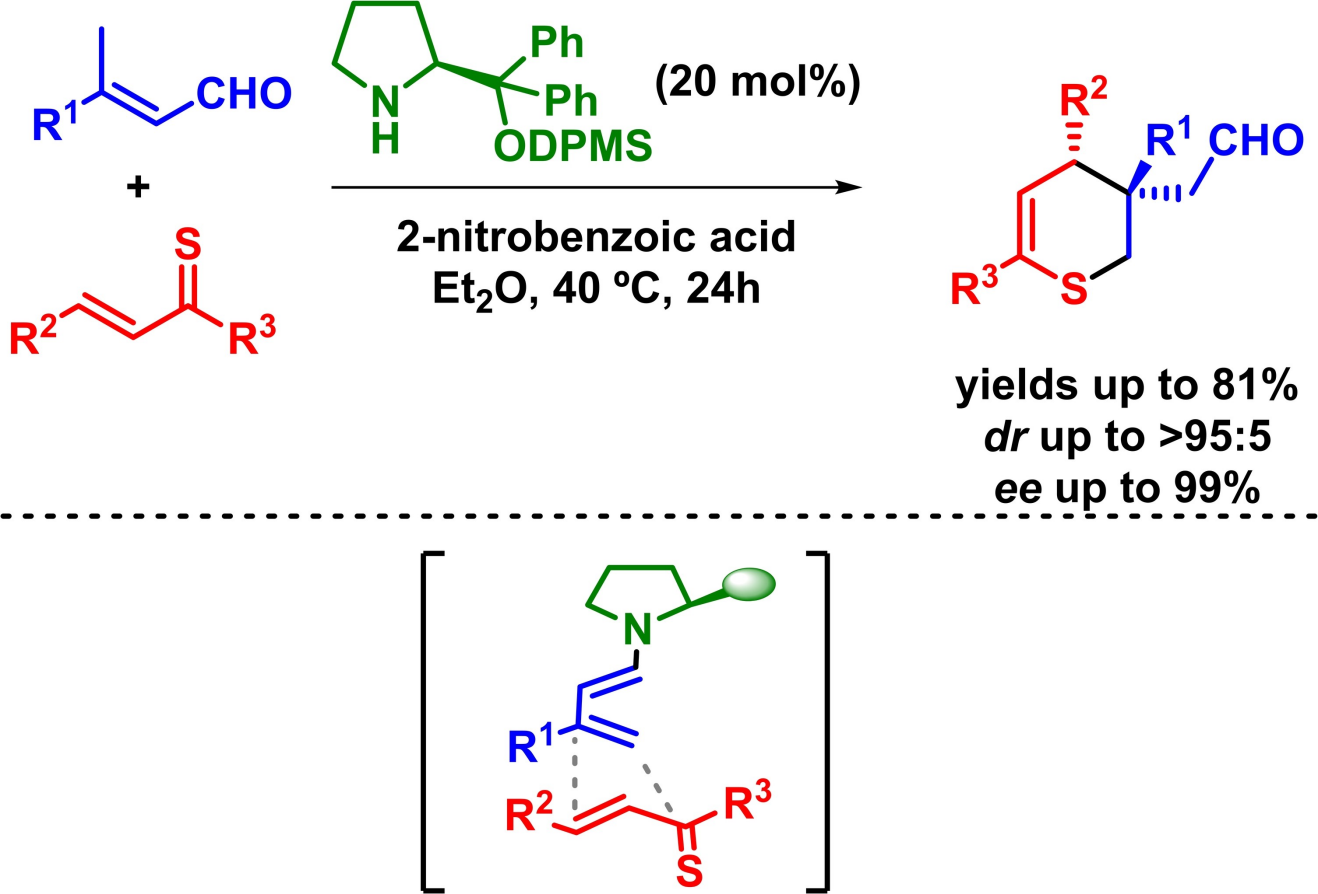
Asymmetric synthesis of 3,4‐dihydro‐*2H*‐thiopyran derivatives through IEDHDAR.

Another example behind this strategy was developed by Albrecht and coworkers in 2017. In this study, the authors described the enantioselective synthesis of 3,4‐dihydro‐*2H*‐thiopyran derivatives by means of an organocatalytic, *ortho*‐regioselective inverse‐electron‐demand hetero‐Diels‐Alder reaction (based on similar previous works).[^29a^, ^29b^] The reaction proceeds with excellent yields, diastereoselectivities and enantioselectivities through and *endo*‐selective mechanistic pathway (Scheme 18).^[29c]^ The authors postulate that after a condensation/deprotonation/isomerization process the generated dienophilic dienamine reacts through its *s*‐*cis* conformation in an *ortho*‐regioselective IEDHDA reaction.

**Scheme 19:**
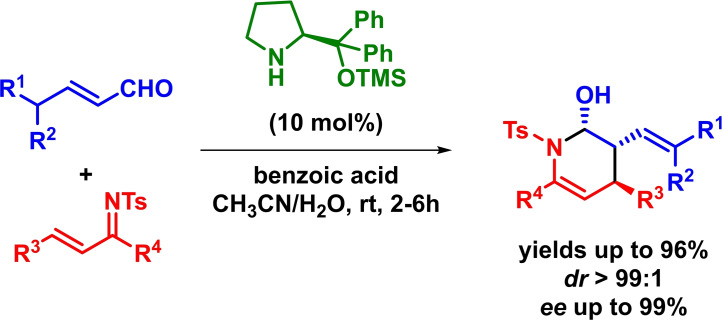
Asymmetric organocatalytic synthesis of hemiaminals via IEDHDAR.

Additionally, Chen also reported the amine‐catalyzed enantioselective inverse‐electron‐demand *aza*‐Diels‐Alder reaction between highly reactive *N*‐tosyl‐1‐aza‐1,3‐butadienes and *α*,*β*‐unsaturated aldehydes. Surprisingly, the authors report that the dienamine presents an unusual *ipso*,*α*‐selectivity, leading to the corresponding chiral hemiaminals in excellent yields, diastereoselectivities and enantioselectivities (Scheme 19).^[30]^

**Scheme 20:**
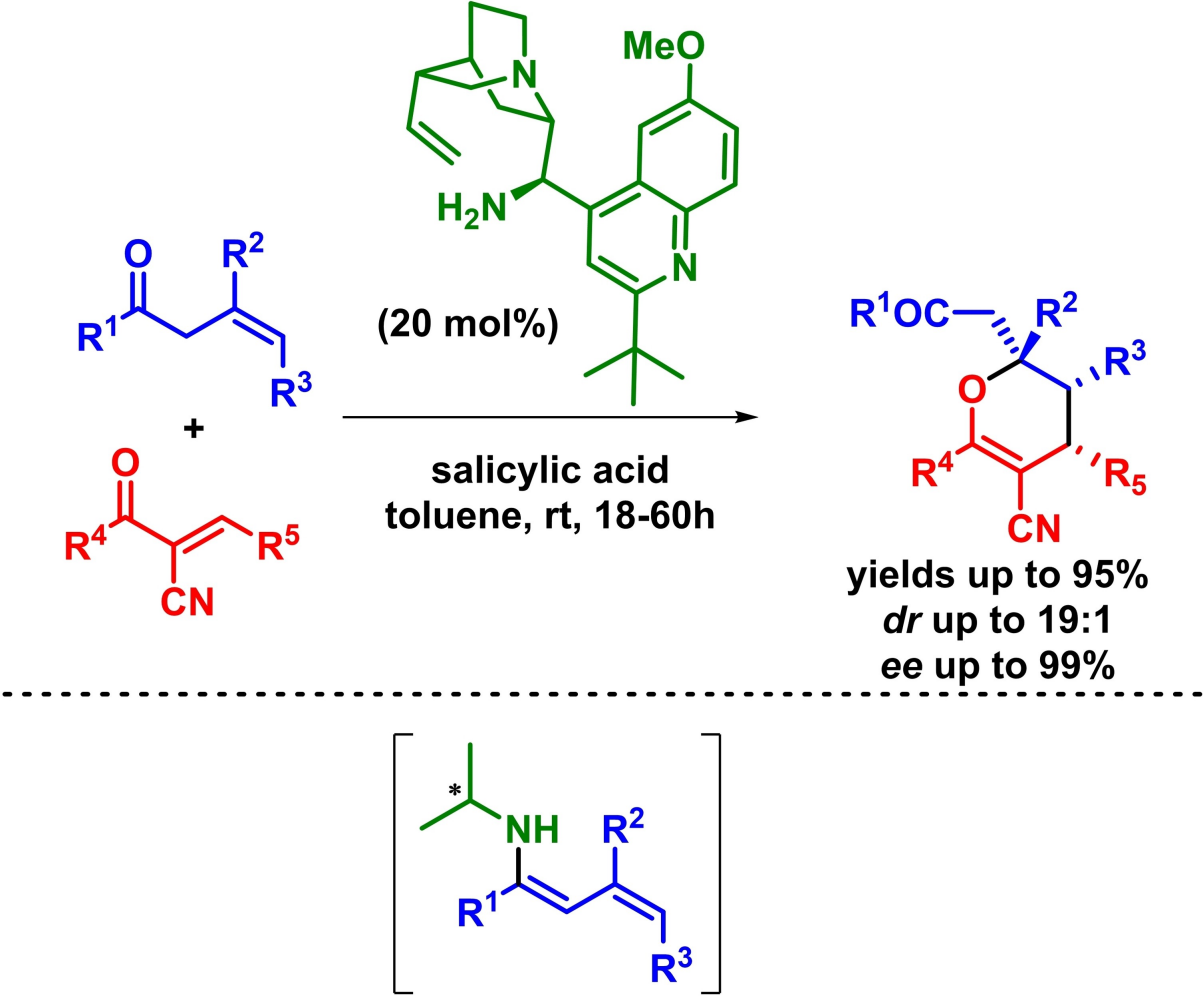
Asymmetric synthesis of dihydropyran derivatives via IEDHDAR.

Furthermore, although aldehydes have been chosen as preferable platforms, ketones have also been included into the reaction portfolio. In this regard, Chen described the use of *β*,*γ*‐unsaturated ketones for the asymmetric electron‐inverse‐demand *oxa*‐Diels‐Alder reaction. The reaction takes place in excellent yields, high diastereoselectivities and excellent enantioselectivities in the presence of a cinchona‐derived primary amine as catalyst. The authors argue that the ketone‐derived in situ formed dienamine evolves through the usual a *β*,*γ*‐regioselectivity, allowing the synthesis of the corresponding highly substituted dihydropyran derivatives (Scheme 20).^[31]^ Concurrently, Pan and coworkers reported a similar methodology, obtaining enantioenriched dihydropyran derivatives with excellent results.^[32]^

## 4 HOMO‐Raising and LUMO‐Lowering Strategies

On the other hand, the use of bifunctional catalysts has been of the significant interest within the organocatalytic field and has largely contributed to the field of asymmetric synthesis.^[33]^ These catalysts, that exploit their capability to perform the simultaneous activation of the electrophile and the nucleophile, have also been employed to synergistically facilitate the HOMO‐raising and the LUMO‐lowering activation of both reaction partners, triggering the asymmetric IEDHDAR.[^6a^, ^6d^, ^21^] This fact has made of this strategy a really interesting approach for the synthesis of compounds that are difficult to obtain by other methodologies.^[34]^ In this sense, the combination of amine catalysis with a hydrogen bond donor core in the same bifunctional organocatalyst has allowed the exploitation of this third strategy through the in situ formation of the corresponding (di)enamine or iminium ion, that would act as dienophile, and the simultaneous activation of the diene through hydrogen‐bonding interactions.

**Scheme 21:**
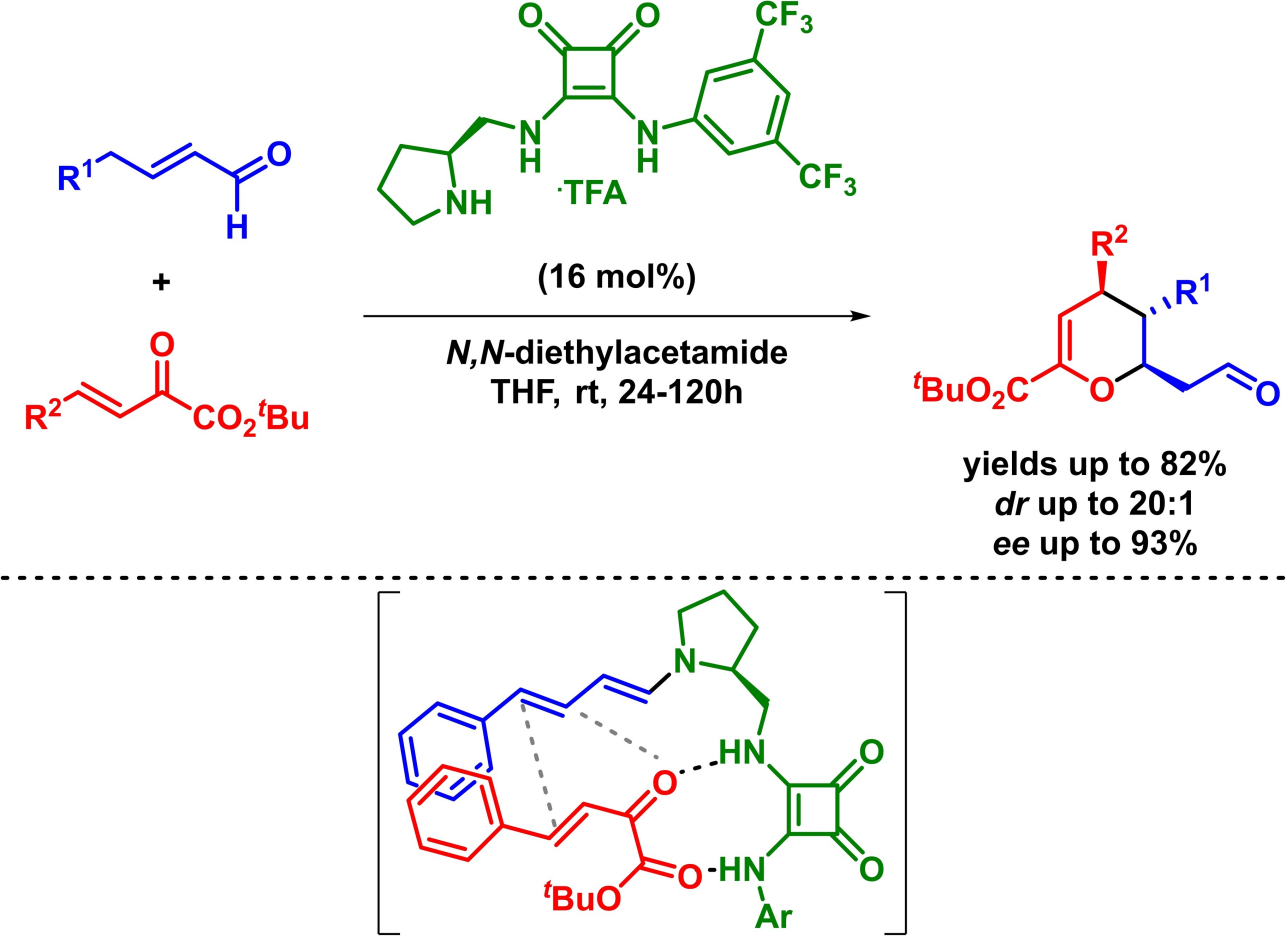
Bifunctional‐catalyzed enantioselective synthesis of dihydropyran derivatives via IEDHDAR.

In this context, Jørgensen described the enantioselective synthesis of dihydropyran derivatives bearing three contiguous stereogenic centers was achieved in excellent yields, high diastereoselectivities and excellent enantioselectivities (Scheme 21). The method is based on a squaramide‐based organocatalyst that bears a pyrrolidine moiety which is involved in the in situ generation of the dienamine (dienophile). The reaction takes place through the expected *β*,*γ*‐regioselectivity and the authors report that the reaction undergoes via a concerted mechanism.^[35]^

**Scheme 22:**
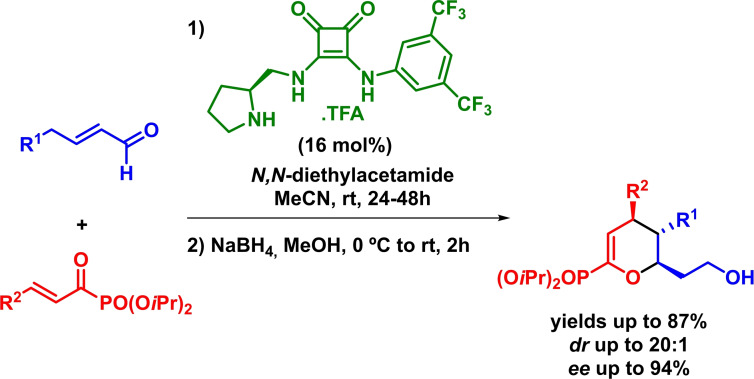
Bifunctional‐catalyzed enantioselective synthesis of dihydropyrans via IEDHDAR.

Some years later, and taking advance of their own previous strategy,^[35]^ Jørgensen developed the enantioselective synthesis of dihydropyran phosphonates. The reaction proceeds with high yields, diastereoselectivities and enantioselectivities in the presence of a wide range of *α*,*β*‐unsaturated acyl phosphonates and of *α*,*β*‐unsaturated aldehydes. Furthermore, it is also worth to mention that the reaction displays the usual *β*,*γ*‐regioselectivity (Scheme 22).^[36]^

**Scheme 23:**
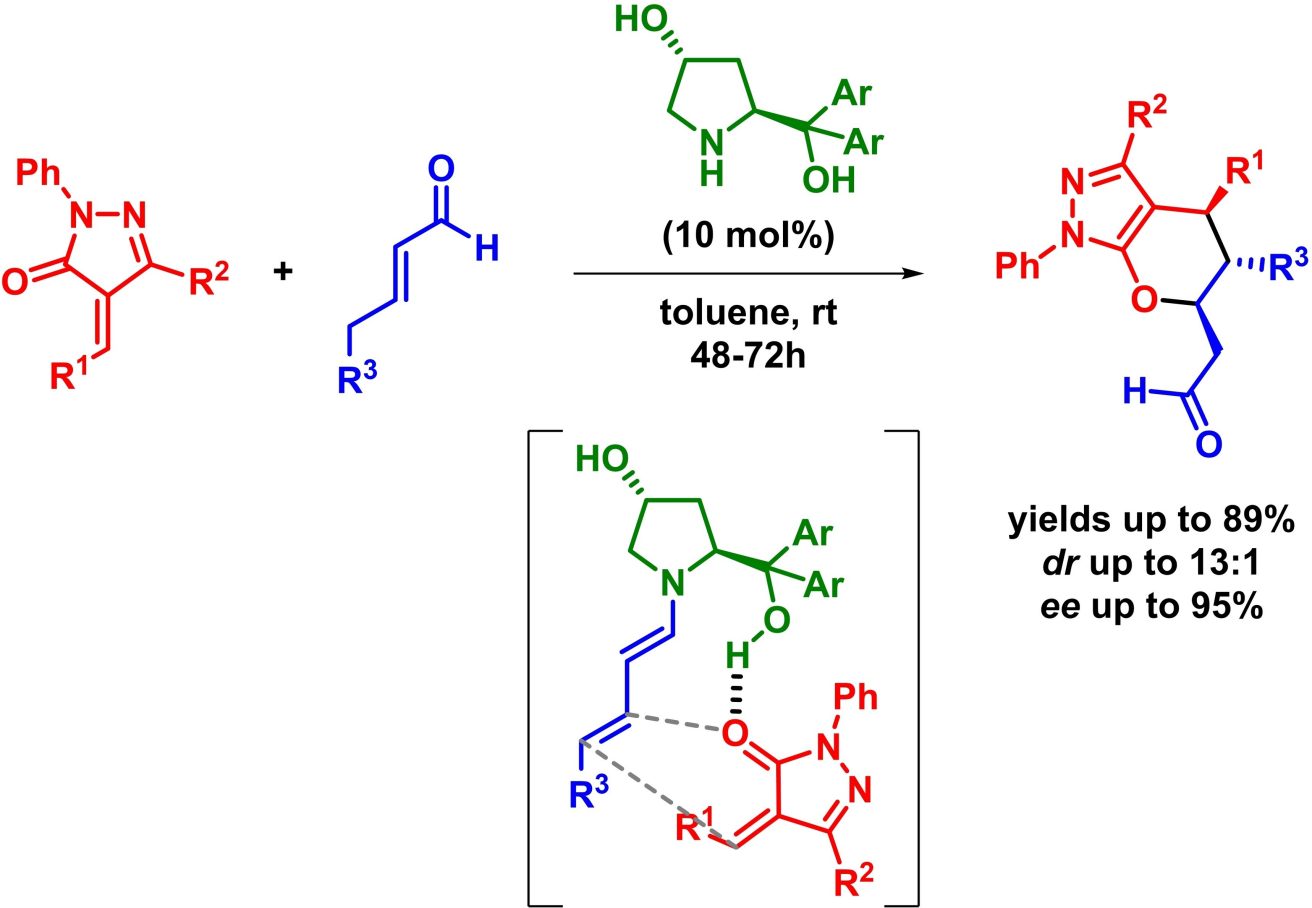
Asymmetric IEDHDAR between enals and pyrazolones.

Two years later, Pericàs and coworkers reported the asymmetric synthesis of tetrahydropyranopyrazole derivatives through a bifunctional‐organocatalyzed inverse‐electron‐demand *oxa*‐Diels‐Alder reaction. The reaction takes place in the presence of differently substituted enals and alkylidene pyrazolones with excellent results (Scheme 23). Moreover, the authors claim that the reaction undergoes through both a concerted mechanism and an *exo* approach in which there is a hydrogen bond between the hydroxyl group present in the aminocatalyst and the substrate.^[37]^

**Scheme 24:**
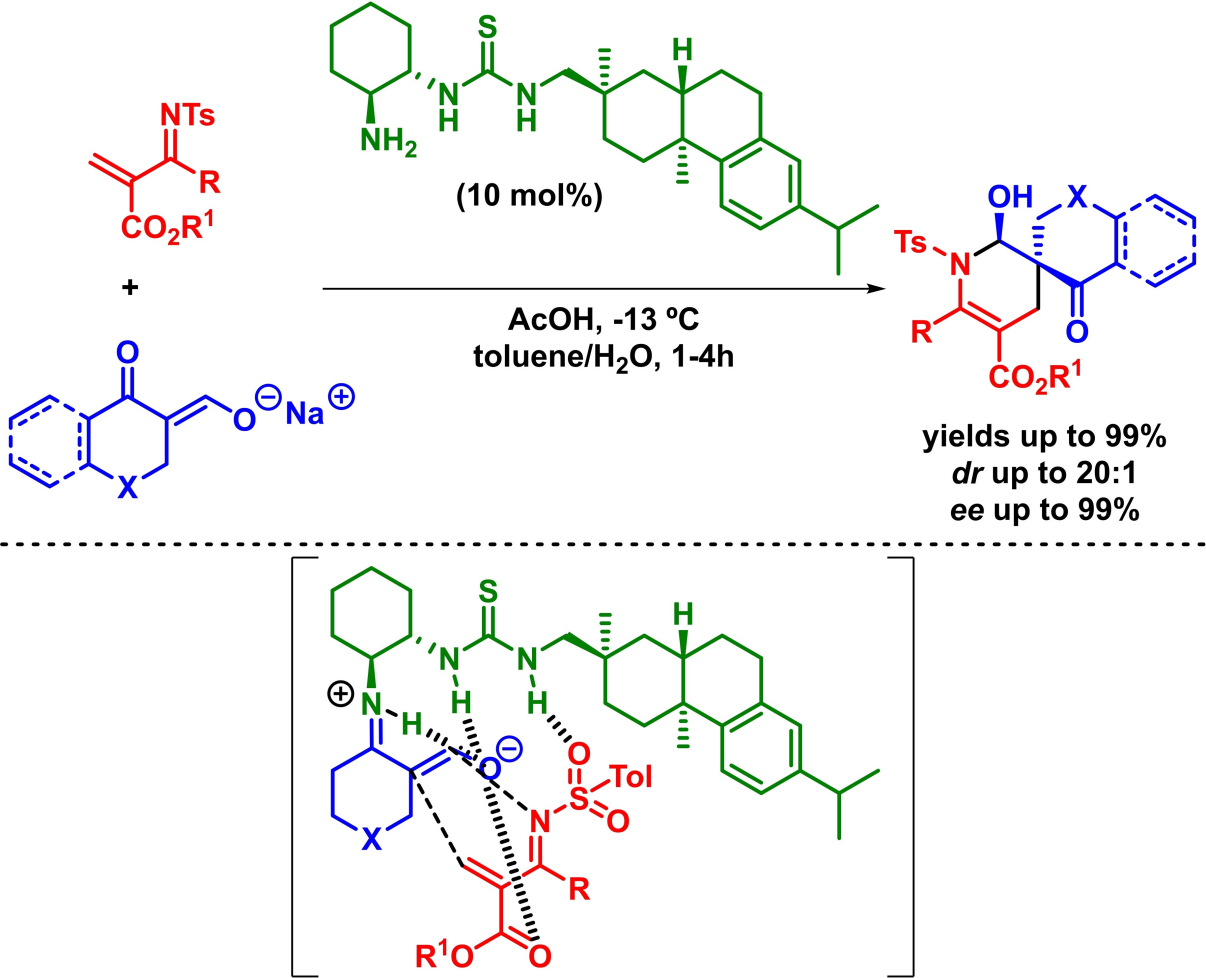
Bifunctional‐catalyzed asymmetric synthesis of azaspirocyclic compounds via IEDHDAR.

In addition, Wang developed the asymmetric synthesis of highly substituted azaspirocyclic compounds. In this case, the reaction is governed by the simultaneous formation of the ketiminium cation of the cyclic keto‐enolates (HOMO‐raising) and the hydrogen bond activation of the *a*‐substituted *α*,*β*‐unsaturated ketimines (LUMO‐lowering). The reaction proceeds with excellent yields, diastereoselectivities and enantioselectivities (Scheme 24). Moreover, the authors propose a mechanistic pathway implicating an *endo*‐selective concerted mechanism in which the attack takes place through the *Re* face.^[38]^

**Scheme 25:**
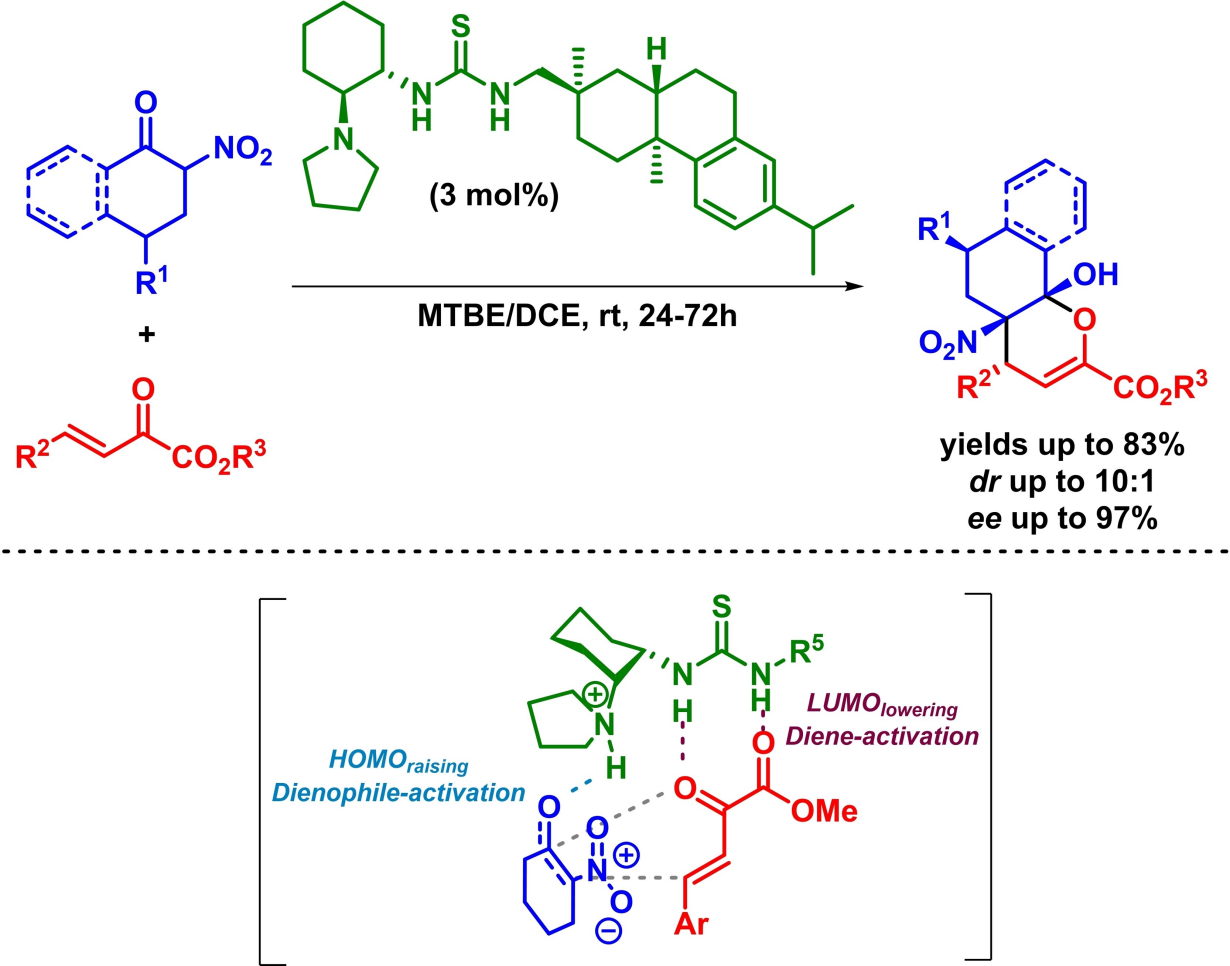
Bifunctional‐catalyzed enantioselective synthesis of bi‐ and tri‐cycles via IEDHDAR.

On another hand, the use of bifunctional catalysts based on both a hydrogen bond donor moiety and a Brønsted base has allowed the simultaneous activation of the electrophile and the deprotonation of the most acidic *α*‐position of different carbonylic compounds, which incorporates new substrates to the IEDHDA reaction collection. The deprotonation process leads to a protonated tertiary amine bearing catalyst, which would be then capable to also activate the dienophile (enolate) as hydrogen bond donor, directing the stereoselective process. In this context, Wang and coworkers reported the enantioselective synthesis of chiral bicycles via a bifunctional‐catalyzed asymmetric IEDHDAR between *α*,*β*‐unsaturated *α*‐ketoesters and cyclic *α*‐nitroketones. The reaction takes place with high yields, moderate diastereoselectivities and high enantioselectivities in the presence of low loadings of a bifunctional organocatalyst (Scheme 25). In addition, the authors argue that the reaction undergoes through a concerted mechanism.^[39]^

**Scheme 26:**
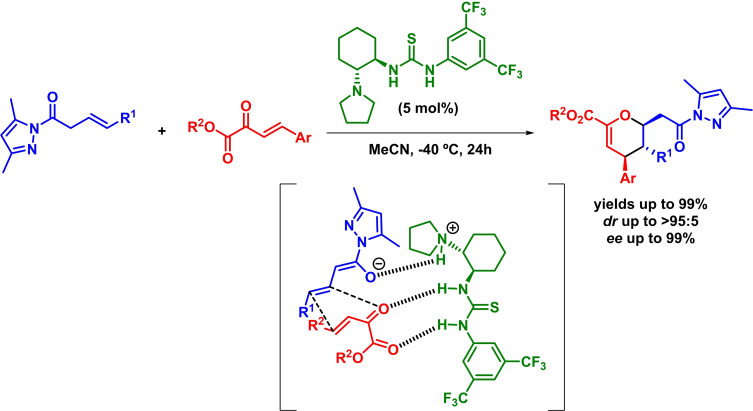
Bifunctional‐catalyzed asymmetric synthesis of dihydropyran derivatives via IEDHDAR.

This strategy has also been extended to the use of *β*,*γ*‐unsaturated compounds as an alternative methodology for the activation of the farthest double bond of the in situ formed dienolate derived from ketones and amides. However, the use of *β*,*γ*‐unsaturated compounds is required when the IEDHDAR takes place following and HOMO‐raising and LUMO‐lowering approach in the presence of this type of bifunctional organocatalysts, as the tertiary amine present in the catalyst is not capable to deprotonate less acidic *γ*‐protons.^[40]^ In this context, Huang developed the reaction between *β*,*γ*‐unsaturated amides and *α*,*β*‐unsaturated *α*‐ketoesters. The reaction takes place in excellent yields, diastereoselectivities and enantioselectivities in the presence of a thiourea‐based bifunctional catalyst (Scheme 26). In addition, a classical *β*,*γ*‐regioselectivity is shown and a stepwise mechanism is postulated.^[41]^

**Scheme 27:**
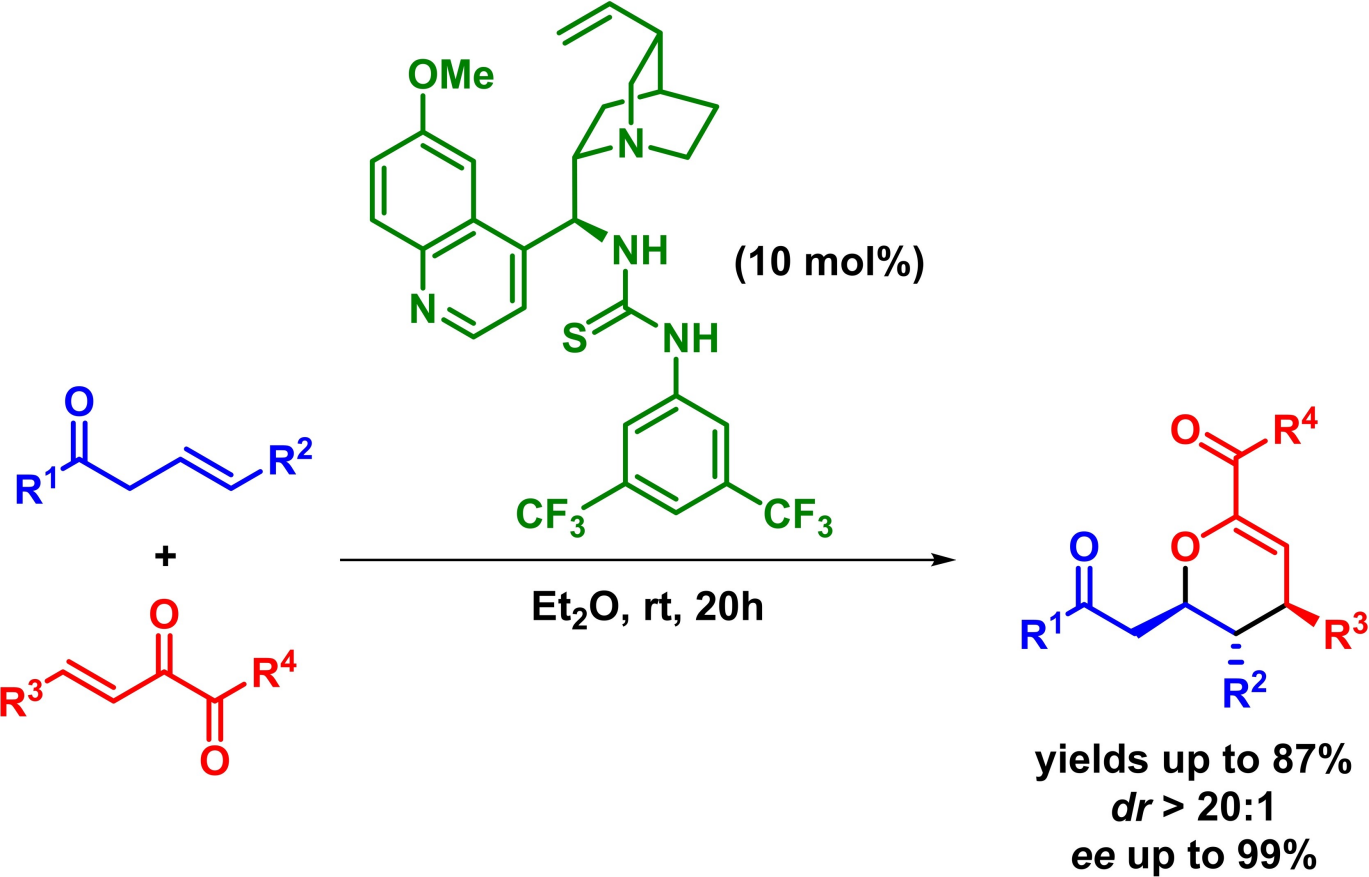
Bifunctional‐catalyzed asymmetric synthesis of dihydropyrans via IEDHDAR.

Simultaneously to the previous work, Yang and Fang reported a similar methodology for the enantioselective synthesis of highly substituted dihydropyran derivatives through a bifunctional‐organocatalyzed inverse‐electron‐demand *oxa*‐Diels‐Alder reaction between *γ*‐substituted *β*,*γ*‐unsaturated ketones and *α*,*β*‐unsaturated 1,2‐diketones. The reaction proceeds with excellent results, showing a *β*,*γ*‐regioselectivity regarding the in situ formed dienolate (Scheme 27).^[42]^

**Scheme 28:**
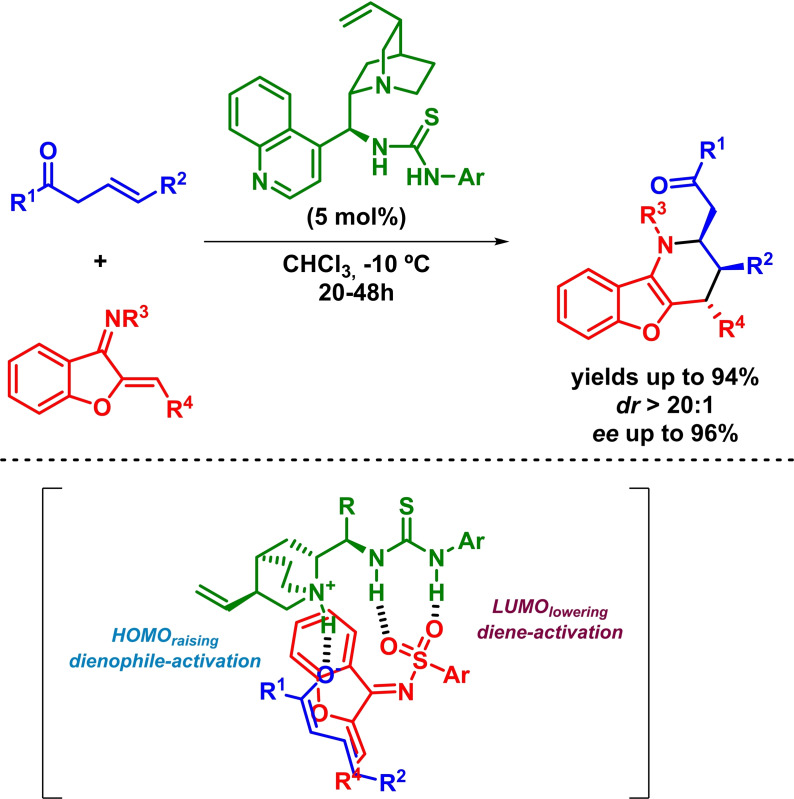
Bifunctional‐catalyzed enantioselective synthesis of benzofuran derivatives via IEDHDAR.

In addition, Albrecht and coworkers have recently described the enantioselective synthesis of benzofuran derivatives bearing a tetrahydropyridine moiety. The reaction takes place in high yields and excellent diastereoselectivities and enantioselectivities in the presence of a thiourea‐based bifunctional catalyst (Scheme 28). In this example, the authors claim that the reaction takes place via a stepwise mechanism. It is also worth to mention that a *β*,*γ*‐regioselectivity is reported as a consequence of the most nucleophilic *γ*‐position of the in situ formed dienolate.^[43]^

**Scheme 29:**
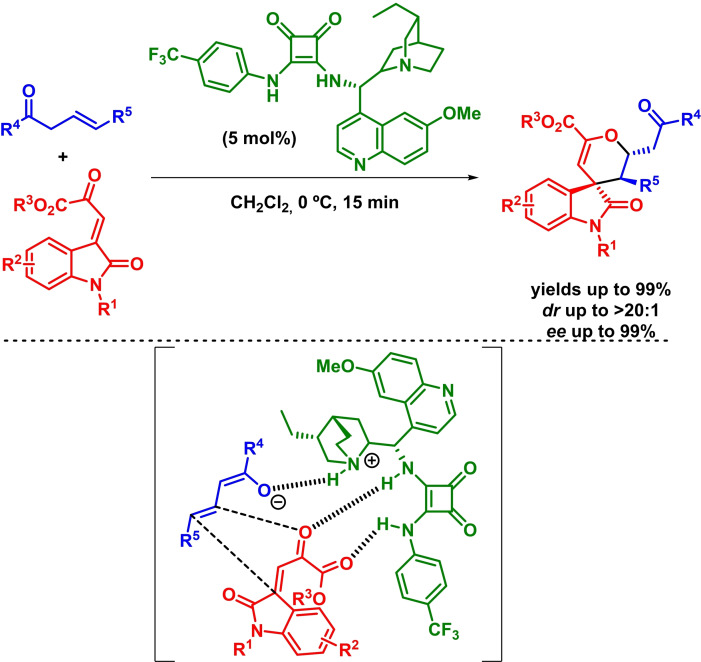
Asymmetric synthesis of 3,4’‐pyranyl spirooxindole derivatives via IEDHDAR.

Another recent example has been released by Zu and coworkers. In this report, the enantioselective synthesis of 3,4’‐pyranyl spirooxindole derivatives with three contiguous chiral centers in their structure were achieved via deprotonation of *β*,*γ*‐unsaturated *α*‐keto esters (Scheme 29).^[44]^

## 5 Summary and Outlook

As a conclusion, organocatalysis is a very well settled research area in organic chemistry, as it has been demonstrated to be an efficient tool in asymmetric synthesis, even for the construction of complex enantioenriched molecular skeletons. We believe that the inverse‐electron‐demand hetero‐Diels‐Alder topic remains a growing field, since it allows the synthesis of structurally diverse compounds that cannot be prepared by other methodologies in a straightforward manner. As shown in this review, a significant number of researchers have smartly employed organocatalysis to develop new inverse‐electron‐demand hetero‐Diels‐Alder type reactions. Several strategies and substrates have been employed for the asymmetric synthesis of six‐membered ring heterocycles using different type of activation modes allowed by the carefully employment and design of new organocatalysts. However, several challenges remain to be addressed in this area. We believe that the major development of this area must come from the discovery of new organocatalysts that incorporate new functionalities able to unlock new activation modes, allowing other substrates, rather than mostly carbonyl derivatives, to be incorporated in the inverse‐electron‐demand hetero‐Diels‐Alder reaction portfolio. This evolution would potentially lead to the synthesis of new families of potential biologically active compounds, enhancing the results yet obtained and even making these new approaches attractive for industrial purposes.

## Conflict of interest

The authors declare no conflict of interest.

## Biographical Information

*Víctor Laina‐Martín was born in Madrid, Spain in 1993. He received his BS in Chemistry in 2015, his MS in Organic Chemistry in 2016 and his PhD in 2021 from the Universidad Autónoma de Madrid (UAM). His research interests are focused on the development of new asymmetric organocatalytic and photocatalytic systems*.

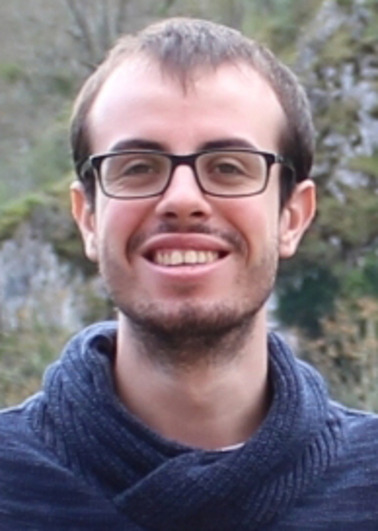

## Biographical Information

*Jose A. Fernández Salas obtained his PhD in 2012 under the supervision of Prof. Jose Luis Garcia Ruano. He then spent 2 years under the supervision of Prof. Steven Nolan at St. Andrews University followed by 2 years under the supervision of Prof. David Procter at University of Manchester, both as postdoctoral researcher. He returned to Spain in 2017 as Juan de la Cierva researcher and he is currently Ramón y Cajal researcher. His research interests include sulfur chemistry, catalysis and asymmetric synthesis*.

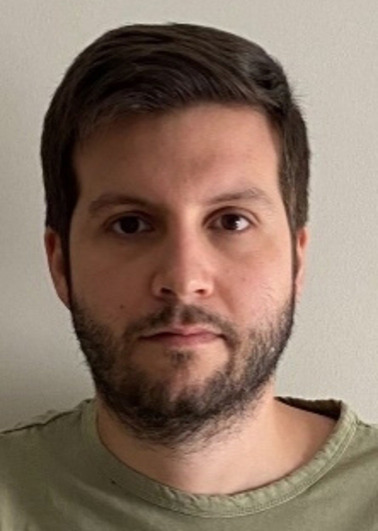

## Biographical Information

*José Alemán obtained his Ph.D. working on sulfur chemistry, under the supervision of Prof. García Ruano in 2005. In 2003, he spent six months in the laboratory of Prof. Albert Padwa at Emory University, Atlanta. Then, he carried out his postdoctoral studies (2006–2008) at the Center for Catalysis in Aarhus (Denmark) with Prof. Karl A. Jørgensen. He returned to Spain in 2009 as a Ramón y Cajal Researcher and is currently Professor at the Universidad Autónoma de Madrid (Spain). He is authour of 165 publications and his research interests include asymmetric synthesis and catalysis*.